# Basement membrane properties and their recapitulation in organ-on-chip applications

**DOI:** 10.1016/j.mtbio.2022.100301

**Published:** 2022-05-23

**Authors:** Golestan Salimbeigi, Nihal E. Vrana, Amir M. Ghaemmaghami, Pinar Y. Huri, Garrett B. McGuinness

**Affiliations:** a School of Mechanical and Manufacturing Engineering, Dublin City University, Dublin 9, Ireland; b Spartha Medical, 14B Rue de la Canardiere, 67100, Strasbourg, France; c National Institute of Health and Medical Research, INSERM UMR1121, Biomaterials and Bioengineering, 11 Rue Humann, 67000, Strasbourg, France; d Immunology & Immuno-bioengineering Group, School of Life Sciences, Faculty of Medicine & Health Sciences, Nottingham, University of Nottingham, NG7 2RD, UK; e Ankara University, Faculty of Engineering, Department of Biomedical Engineering, Ankara, 06560, Turkey

## Abstract

Drug discovery and toxicology is a complex process that involves considerable basic research and preclinical evaluation. These depend highly on animal testing which often fails to predict human trial outcomes due to species differences. Coupled with ethical concerns around animal testing, this leads to a high demand for improved *in vitro* cell culture platforms. Current research efforts, in this regard, however, are facing a challenge to provide physiologically relevant *in vitro* human organ models for a reliable assessment of the physiological responses of the body to drug compounds and toxins. The latest development in *in vitro* cell culture models, organ-on-chips (OOCs), seek to introduce more realistic models of organ function. Current OOCs often use commercial porous polymeric membranes as a barrier membrane for cell culture which is challenging due to the poor replication of the physiological architectures. Better recapitulation of the native basement membrane (BM) characteristics is desirable for modelling physical (e.g. intestine, skin and lung) and metabolic (e.g. liver) barrier models. In this review, the relevance of the physical and mechanical properties of the membrane to cell and system behaviour is elucidated. Key parameters for replicating the BM are also described. This review provides information for future development of barrier organ models focusing on BM-mimicking substrates as a core structure.

## Graphical abstract

**Figure:**
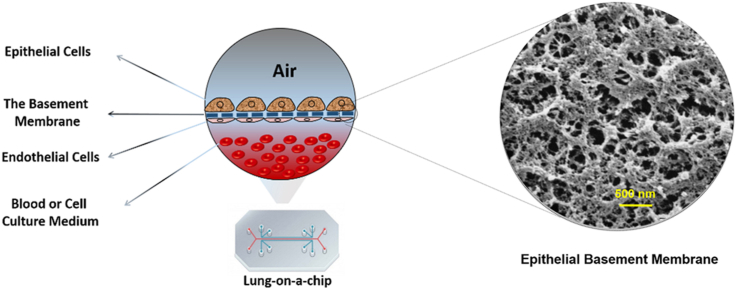

## 1 Introduction

Current drug development and toxicology research are facing a challenge with regards to reliable predictive models to assess physiological responses of tissues to drugs and chemicals. To increase the success rate of toxicity and efficacy prediction for drugs and engineered biomimetic tissue models, developing new robust cell culture platforms is paramount. Simulation of the biological, mechanical, physiological, or pathological cellular microenvironment is necessary for developing functional *in vitro* models which are key elements of basic research, disease modelling, drug discovery, and tissue replacement [[1], [2], [3]].

Animal models, however, are still considered the main source of data collection in early drug development studies for predicting human pharmacokinetic responses as they capture the complex physiology of *in vivo* tissues, with cells residing in a dynamic environment continuously being perfused with blood. Cells *in vivo* benefit from interactions with other cells and they are stimulated by chemical, mechanical and electrical cues [[4], [5], [6]].

Pre-clinical animal studies, however, are too costly and are often inaccurate as successful testing in animals does not guarantee successful results in human trials. This is largely due to species-specific differences including metabolic and physiological differences. Despite considerable similarities in genetic and physiological characteristics between animals and humans, animal models often fail to predict drug performance accurately in humans. Moreover, there are also ethical concerns with the use of animals for this purpose [7].

Along with these limitations, the reaction of patients to drugs can greatly differ as the entire spectrum of responses to a drug is not covered by these clinical trials. The trials before drug approval are based on the selection of patients on characteristics such as age or sex and therefore, they do not represent the entire population. Children, in particular, are generally not considered due to ethical concerns [8].

The limitations related to animal models have led to greater demands for *in vitro* alternatives recapitulating the structural and functional complexity of living tissues and organs. Conventional two-dimensional (2D) cell culture platforms benefit from easy handling, low cost, easy microscopic visualization, good quantity and purity of cellular extract and are useful for providing information on early biological responses and effective for high-throughput drug screening. However, they often fail to reconstitute the *in vivo* cellular microenvironment and show limited predictive capabilities [9,10].

Offering three-dimension (3D) spatial stimulation and a better resemblance to the extracellular matrix (ECM), 3D scaffolds with a higher capability of capturing complex physiological responses have emerged. Artificial 3D cell culture models such as hydrogels or fibrous scaffolds utilize biocompatible polymer materials or natural biological extracts to provide an ECM-like environment for culturing cells. These approaches induce differentiated tissue-specific phenotypes and improve tissue organization. Cell infiltration into fibrous scaffolds can be challenging due to their compact structure and small pore size. This lack of cell penetration can prevent appropriate tissue remodelling, especially for complex tissues such as the heart and liver [[11], [12], [13], [14], [15]]. *In vitro* organ models featuring cell co-culture are not generally standardised for high or low throughput automation or optimized in terms of consumables and labour.

These shortcomings have led researchers toward developing the next generation of advanced cell culture models, OOCs. Relying on developments in biomaterials, biology, micro-machining, microfluidics and biofabrication, these advanced systems enable the recreation of structural, environmental and functional properties of complex human organs. Combining micro-engineering technologies with cultured cells, these microfluidic devices are capable of recapitulating the physiological and mechanical microenvironment of living organs [13,[16], [17], [18]].

OOCs technology has the potential to revolutionize drug development by offering specialized *in vitro* tissue models as miniaturized platforms for conducting drug testing studies. Providing more physiologically relevant conditions and a dynamic culture environment by perfusion of the media in a laminar flow, they enable the efficient study of cellular behaviour against various physical and chemical stimuli. Moreover, the dynamic cellular responses can be monitored by incorporating biosensors and electrodes into a single device. They benefit from high spatiotemporal precision utilising microfluidics which offers control over chemical and physical microenvironments of the cells [8,[19], [20], [21]].

**Fig. 1:**
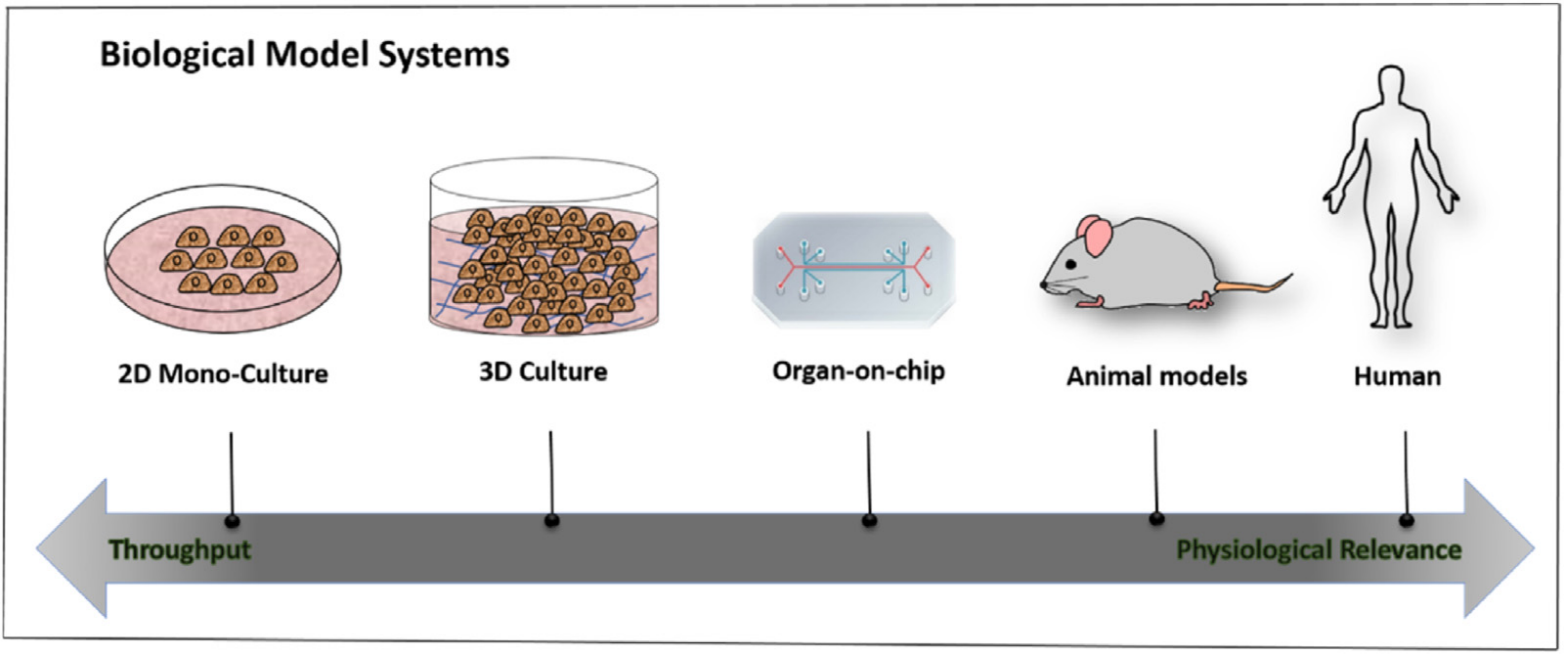
Biological model systems spectrum in terms of their experimental tractability and physiological relevance.

Various state of the art OOCs platforms have been developed by researchers including for example vessel-on-chip [22,23], liver-on-chip [24,25], heart-on-chip [26], tumour-on-chip, FBR (foreign body response)-on-chip [27], muscle-on-chip [28,29], intestine-on-chip [[30], [31], [32]] and lung-on-chip [[33], [34], [35], [36], [37]], although this is not a complete list. Multi-organ-on-chip is also rapidly developing leading researchers toward creating a human-on-chip system comprised of various organ-on-chips that can be completed with blood circulation [1,[38], [39], [40]]. Fig. 1 summarizes the biological model systems placed on a spectrum in terms of their experimental tractability and physiological relevance.

Nevertheless, one of the limitations of currently available OOCs is the lack of physiologically relevant membranes, with patterned flat polymeric membranes often being used. The membrane is an integral part of the chip which significantly affects cellular behaviour. Current systems are mainly based on synthetic porous elastic polydimethylsiloxane membrane (PDMS) which do not attempt to replicate the extracellular matrix (ECM) [41]. Cells *in vivo* are surrounded by the ECM. A proper recapitulation of the chemical and physical complexity of ECM is ultimately necessary for the representative performance of cells *in vitro* [42].

**Fig. 2:**
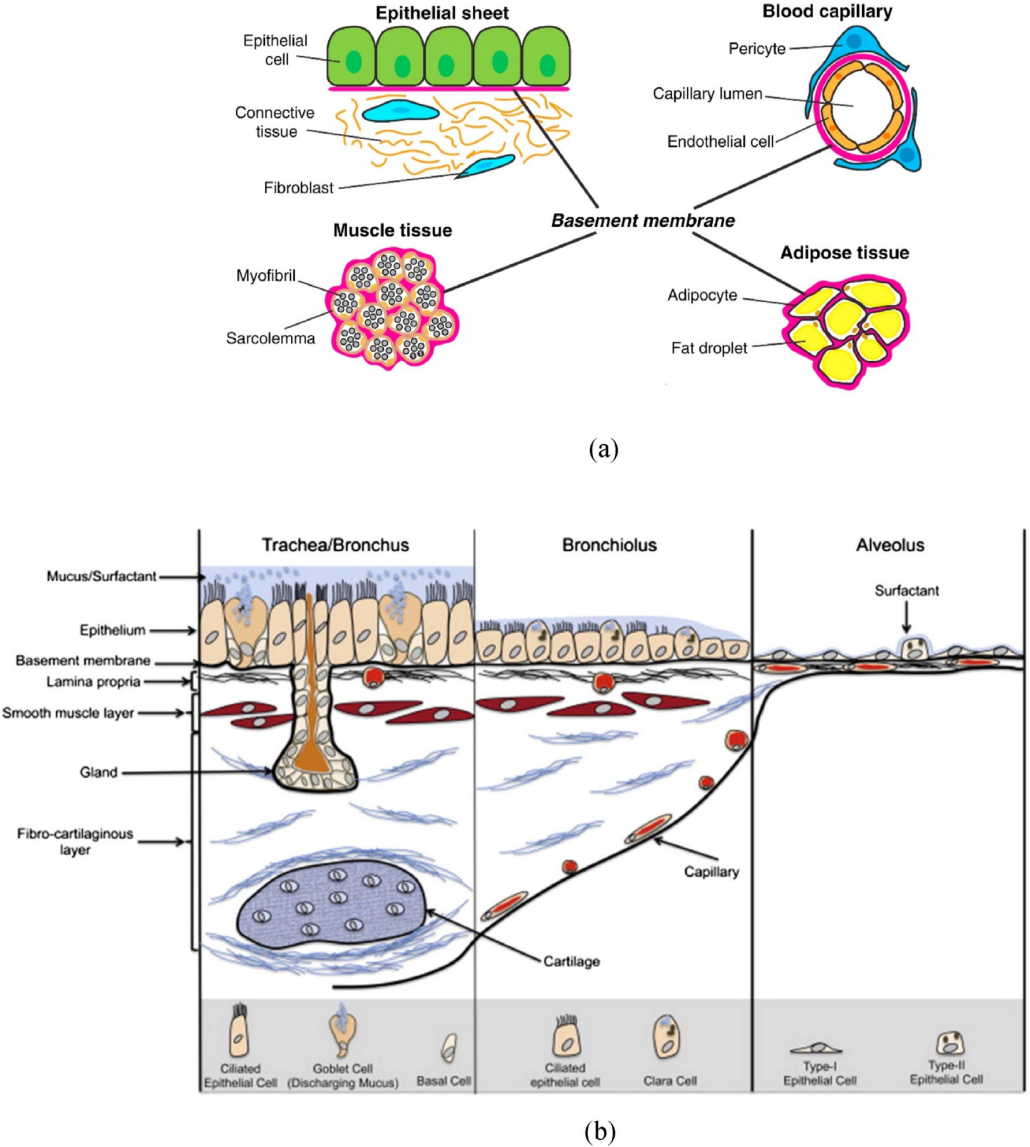
a) BMs support or surround epithelial, endothelial, muscle, and adipose tissues [46] Reprinted from Current Biology, Vol 27, Ranjay Jayadev, David R. Sherwood, Basement Membranes, R207-R211., Copyright (2017), with permission from Elsevier, b) Throughout the respiratory tract, the cell type and morphology change in concert with their physiological function [47]. Reprinted from Advanced drug delivery reviews, Hittinger, Marius, Jenny Juntke, Stephanie Kletting, Nicole Schneider-Daum, Cristiane de Souza Carvalho, and Claus-Michael Lehr, Preclinical safety and efficacy models for pulmonary drug delivery of antimicrobials with focus on in vitro models, Vol. 85, Pages No. 44-56, Copyright (2015), with permission from Elsevier.

The BM, a specialized thin sheet-like ECM, is a densified layer separating cell populations from connective tissues. It surrounds most tissues, including epithelial, endothelial, muscle, and adipose tissues (Fig. 2a). Fig. 2b demonstrates the location of the BM and cells within the respiratory system. Apart from its barrier function and providing mechanical support for cells, BM also regulates cellular behaviours such as cell differentiation, proliferation, migration, adhesion, axon growth, and polarization [[43], [44], [45]].

The BM structure varies in thickness and composition depending on its location. However, it mainly is a very complex two-layer structure composed of various proteins such as collagen, fibronectin and laminin. As basement membranes (BMs) are important in the development and functional integrity of tissues, recreating this complex structure is, therefore, necessary to have a successful tissue model [[48], [49], [50]]. Despite many efforts, the reconstruction of tissue models has been less successful due to a lack of topographical, physical, and mechanical recapitulation of the natural ECM. An improvement in OOCs membranes is necessary to have a better physiologically relevant model along with the advancement in its other components.

This review will briefly compare 3D cell culture platforms and OOCs. It then highlights the need for more physiologically relevant OOCs by developing more suitable biomimetic membranes. The topographical, physical and mechanical properties of different BMs are then reviewed. Furthermore, membrane design, candidate materials, and manufacturing processes are also discussed. Eventually, the optimal synthetic BM and the integration of the most frequently used scaffolding materials in microfluidic systems and their challenges will be briefly discussed.

## 2 From 3D cell culture systems to OOCs

*In vitro* cell-culture models can be divided into two categories: static and dynamic. Static systems can be found in planar designs or 3D arrangements. Cells may be grown in a monolayer arrangement in culture wells, dishes or flasks. These systems are the most widely used cell culture systems due to the low cost, simple design, easy control over the process, straightforward microscopic visualization, and good quantity and purity of cellular extracts. However, for certain organs, they resemble the *in vivo* state the least. Static 3D culture systems can offer a spatial simulation of the ECM. Moreover, a sufficiently porous scaffold with high pore interconnectivity can enable improved cell infiltration. Decellularized tissues can also be used as 3D scaffolds, though intrinsic variability and susceptibility to denaturation during decellularization are their main drawbacks [9,10,13,51].

Bioreactors and microfluidic chips, on the other hand, introduce dynamic stimuli to the system that closely recapitulate those experienced by the cells *in vivo*. Bioreactors introduce dynamic culture conditions to 3D scaffolds using pumps. Microfluidic systems, however, handle extremely small fluid volumes that are manipulated in equally small channels (tens to hundreds of microns). The fluid volumes are in the order of 10^−6^ to 10^−18^ ​L [52].

Combining bioreactors with microfluidic channels, microfluidic chips introduce 2 or 3D dynamic cell culture systems with high descriptive power. They can therefore benefit from the advantages offered by both 3D cell culture systems and dynamic culture conditions, mimicking the microenvironment of complex tissue-circulatory system tissue-tissue interfaces. They can provide the mechanical and chemical cues provided by the dynamic microenvironment [53].

OOCs can reduce the costs of drug development [[54], [55], [56]] which can exceed $800 million for a single drug, only 10–30% of which can make it to the market [57]. Pre-clinical animal studies that are necessary for the process of drug development are too costly and are often inaccurate as successful testing in animals does not guarantee successful results in human trials. This is largely due to species differences. Despite considerable similarities in genetic and physiological characteristics between animals and humans, animal models often fail to predict drug performance accurately in humans. Moreover, there are also ethical concerns with these tests [7].

Although *in vitro* models lack the physiology of tissues as opposed to animal models, the utilization of human cells in these models can lead to a more accurate drug response prediction and they can also account for individual differences [41]. While cell lines are mainly used in OOCs, they have the potential to be combined with human-induced pluripotent stem cells (hiPSCs) resulting in a powerful tool for developing personalised therapies that can also be employed for children [41].

OOCs have great potential for the application of personalised medicine in clinical practice which can lead to an individual-specific evaluation of drug efficacy and safety coupled with personalised strategies for more accurate diagnosis, drug development and, therefore, optimal treatments. Using personal health data to tune key physico-chemical parameters of the cell culture microenvironment together with the inclusion of blood samples and primary human cells makes precision medicine feasible [58,59]. As such, researchers have recently developed a foreign body response (FBR)-on-chip with the potential of monitoring the foreign body response in a personalised manner [27]. Their findings revealed a variation in the differentiation of primary human monocytes into M1 and M2 phenotypes from one donor to another. This highlights the significance of the personalised OOCs systems, particularly FBR-on-Chip due to a significant level of inter-individual variability derived from different immunological profiles [60,61]. FBR is a serious challenge for implants devices and biomaterials [27]. Personalised screening of the FBR to implants can be very promising to estimate and develop strategies to regulate such a response.

**Fig. 3:**
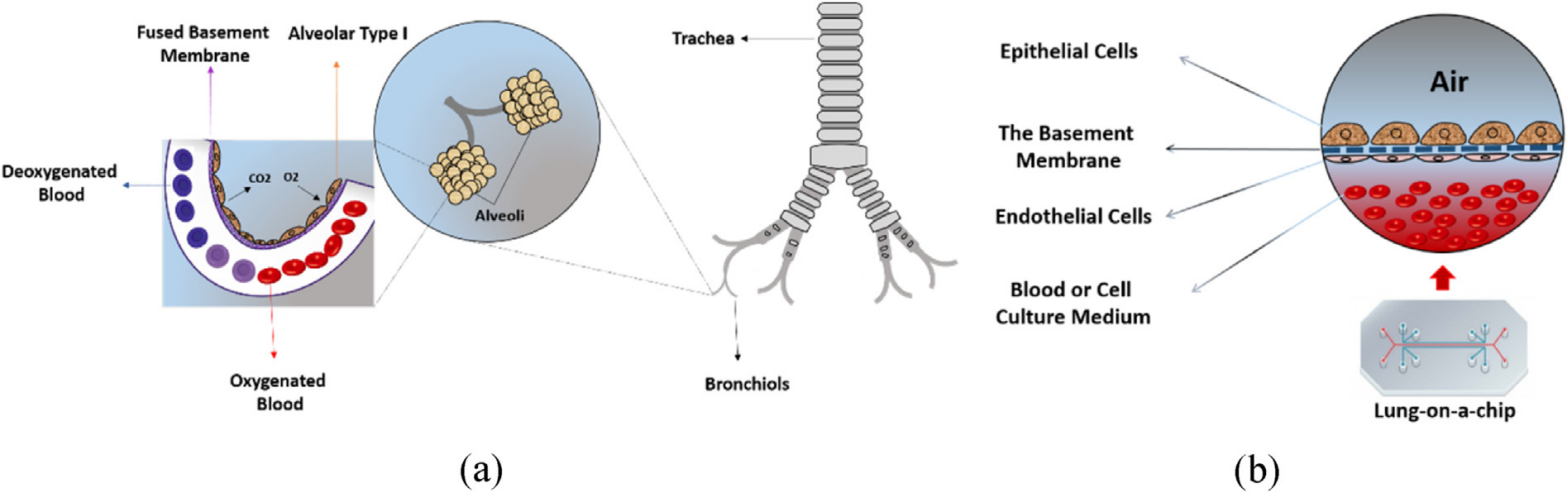
a) Schematic of human respiratory airways and the alveoli, b) Lung-on-chip illustration.

Many of the characteristics of different OOCs are common despite the necessary differences to recapitulate specific organs. They typically consist of a porous polymeric membrane separating a blood vessel compartment containing endothelial cells from an organ compartment containing relevant cells to recreate the essential aspects of the desired organ [41]. The membrane should ideally exhibit the characteristics of the BM, the most important of which are the ability to demarcate different types of cells that grow on opposite sides, structural support, cell adhesion and function and permeability to allow the transport of oxygen, nutrients, other waste materials, and also immune cells. Fig. 3a depicts the structure of the functional region of the lung, the alveoli, while Fig. 3b shows the components of a typical lung-on-chip in detail which recreates the structure of the alveolus.

OOCs are particularly favourable for modelling barrier tissues, such as lung, as they enable physiologic air-liquid interface (ALI) culture conditions and recapitulation of various physiological movements such as breathing motions and liquid plug formation. Mechanical stimuli have a major role in the regeneration of the lung and also significantly affect disease onset and chronicity in pathological conditions [62,63]. Breathing-induced mechanical forces significantly affect cellular behaviours and they need to be considered in any rational lung model design [64,65]. While the important role of mechanical forces to recapitulate the physiological and pathological conditions of the lung is well recognized, most *in vitro* models cannot provide stretch forces to the epithelial cells.

**Fig. 4:**
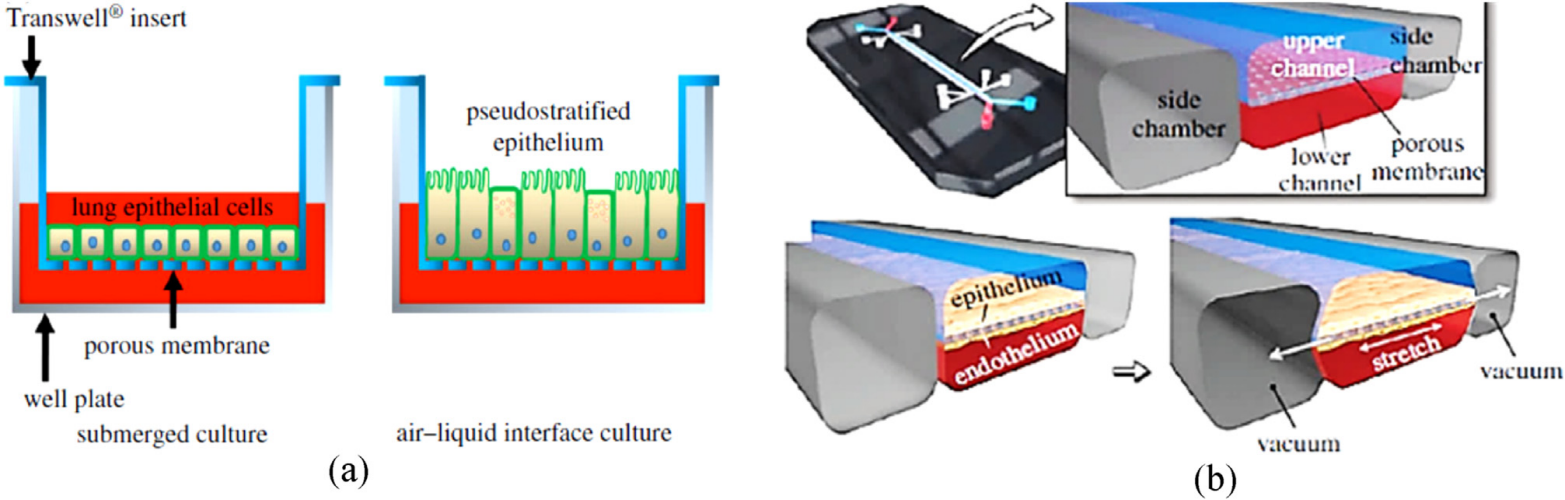
Schematic representation of lung cell culture systems, (a): Lung epithelial cells are cultured in a conventional manner on porous poly (bisphenol-A carbonate) or poly (ethylene terephthalate) (PET) membrane using a commercially available Transwell system, republished with permission of The Royal Society (U.K.), from Flat and microstructured polymeric membranes in organs-on-chips, Pasman, Thijs, Dirk Grijpma, Dimitrios Stamatialis, and Andreas Poot, Volume 15, Issue 144, 2018, permission conveyed through Copyright Clearance Center, Inc. [41]. (b): A lung-on-chip consisting of a microfluidic device with three channels. The middle channel contains two compartments, separated by a porous PDMS membrane. Lung epithelial cells are cultured on the top side, while endothelial cells are cultured on the bottom side of the membrane. Air and cell culture medium flow through the top and bottom compartments, respectively. A vacuum can be applied in the adjacent two channels which provide mechanical stretch to the membrane and cells. Reprinted with permission from Nature Protocols, Huh, Dongeun, Hyun Jung Kim, Jacob P. Fraser, Daniel E. Shea, Mohammed Khan, Anthony Bahinski, Geraldine A. Hamilton, and Donald E. Ingber, Microfabrication of human organs-on-chips, Vol. 8, Issue 11, Pages No. 2135-2157, Copyright (2013) [67].

Furthermore, cells are mainly submerged in cell culture media rather than being exposed to an air-liquid interface, as found in the lung, to enable the secretion of a protective liquid layer resulting from cell polarization [66]. The Lung-on-chip system not only offers a more biomimetic *in vitro* model by controlling microenvironment factors and enabling the mass transfer of nutrients but also can meet the requirements for a stretchable ALI cell culture model. Fig. 4 compares the lung cell culture system (Transwell insert) to a lung-on-chip.

**Fig. 5:**
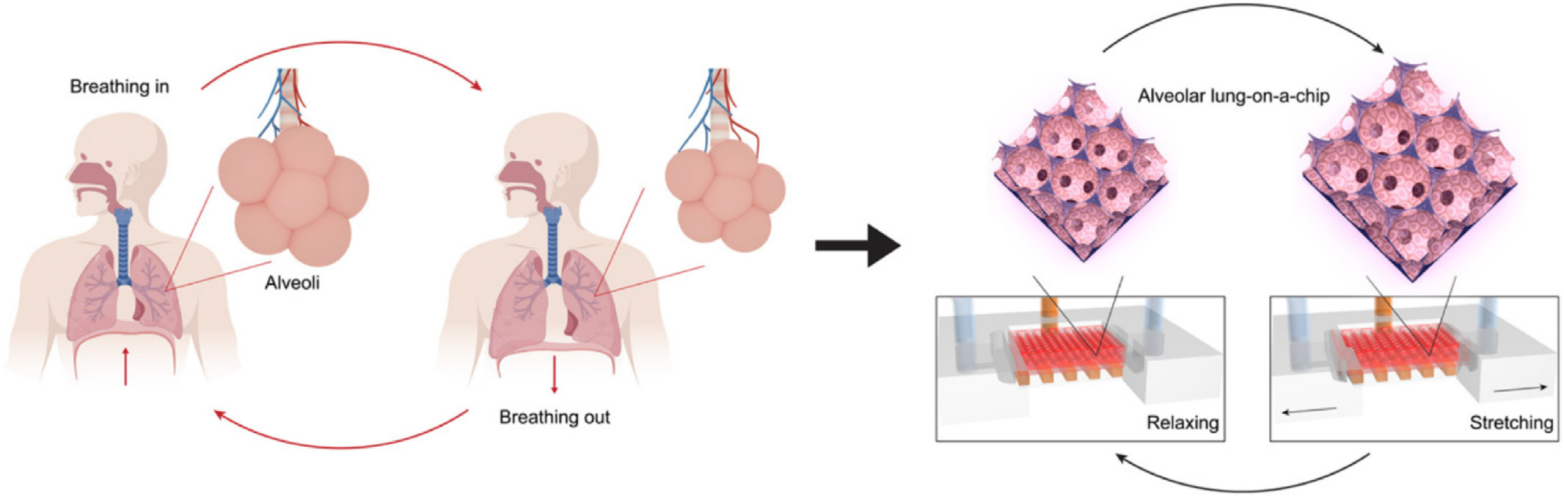
The breathing human alveolar lung-on-chip. Schematics showing the distal lung, the breathing cycles, and the *in vitro* on-chip model of the lung [37]. Republished from Reversed-engineered human alveolar lung-on-a-chip model, Huang, Di, Tingting Liu, Junlong Liao, Sushila Maharjan, Xin Xie, Montserrat Pérez, Ingrid Anaya et al. Proceedings of the National Academy of Sciences 118, no. 19 (2021).

A more advanced and physiologically relevant alveolar lung-on-chip model capable of capturing different aspects of the lung was developed by Huang et al. [37]*.* A 3D porous hydrogel gelatin methacryloyl (GelMA) with an inverse opal structure was fabricated to recreate the microarchitecture of the alveoli. With low stiffness (6.23 ​± ​0.64 ​kPa), sac-like pores and the interconnecting windows between the sacs, the scaffold showed great resemblance to the alveoli. The scaffold was then bonded to a compartmentalised PDMS chip device capable of providing an air-liquid interface and cyclic breathing motions (See Fig. 5). The function of primary human alveolar epithelial cells was better maintained in this system as opposed to planar models due to better recapitulation of the structural and functional features of the human pulmonary alveoli.

## 3 The basement membrane

**Fig. 6:**
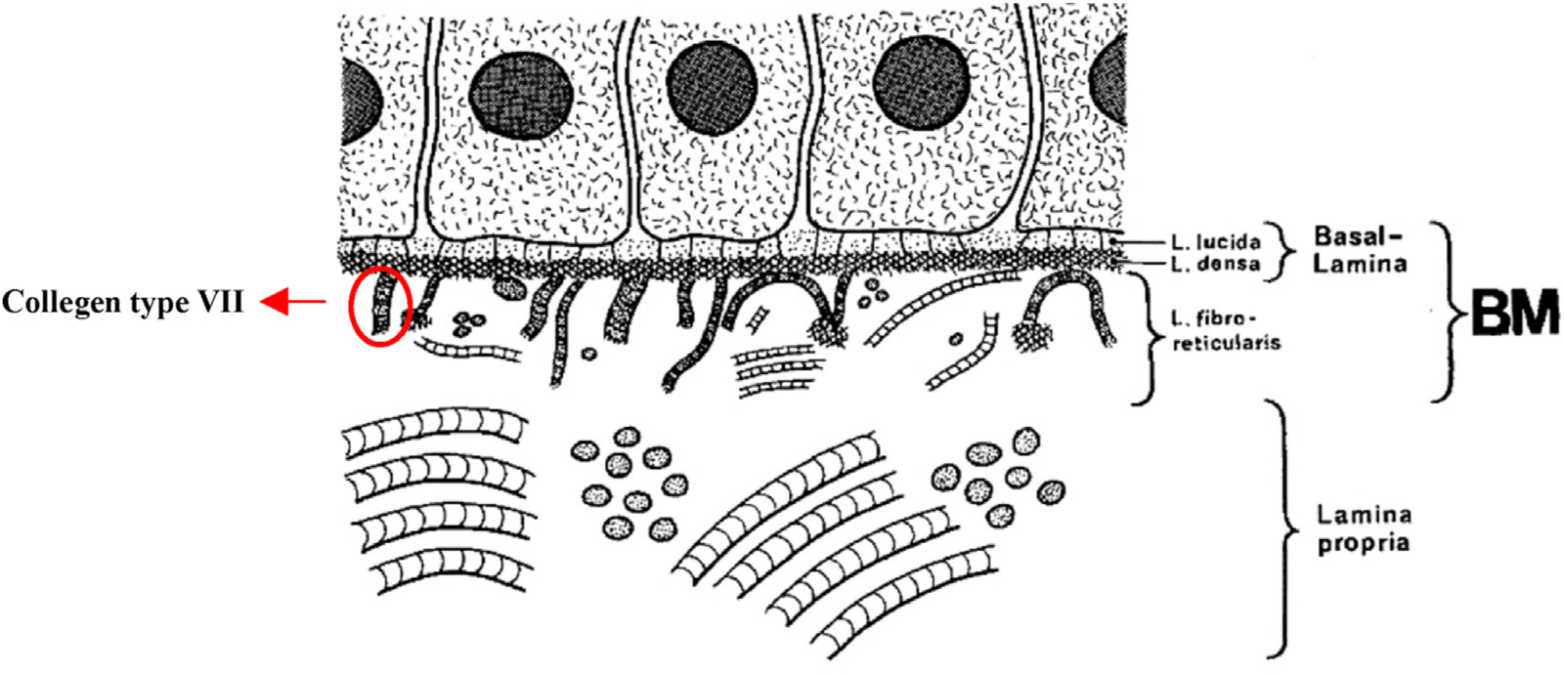
Schematic representation of the structure of the BM: L.l, L.d, and L.f with collagen type VII filaments (anchor filaments) and singly, irregularly running cross-striated collagenous fibrils [48]. Republished with permission of John Wiley & Sons Inc, from Morphology of the Basement Membrane, Merker, Hans-Joachim , Volume 28, Issue 2, 1994; permission conveyed through Copyright Clearance Center, Inc.

Our understanding of the components, morphological structure, functions and genetics of the BM has developed over the years due to advances in many fields and a combination of various approaches including biochemical, biophysical, cell biological, genetic, and bioengineering. Electron microscopically, the components of BM include lamina Lucida (L.l.), lamina densa (L.d.), and lamina fibro reticularis (L.f.) (sometimes it's referred to as reticular lamina or pars fibroreticularis in some references) demonstrated in Fig. 6 [48]. Both lamina lucida and lamina densa form the basal lamina. The reticular lamina is not present in some BM. That is why BM is sometimes referred to as basal lamina [68,69]. It is expected that there is one huge BM that demarcates connective tissue from all parenchyma. In practice, however, the BM can be missing, in the liver for example, or may be interrupted, such as along the intestinal epithelium [70,71].

### 3.1 Basal lamina

#### 3.1.1 Lamina densa (L.d)

L.d is functionally the most important component of the BM and is always present. In fact, the absence of L.d implies a lack of BM. The high magnification of the electron microscopy can reveal an accurate demonstration of the L.d shown as a grey zone. The thickness of L.d varies depending on the location and the tissue origin from 15 to 125 ​nm [69]. BM can be categorized as normal or simple, double, and thick. The presence of two L.d layers referred to as double BM can be observed in areas where the L.d of the epithelium and endothelium meet, e.g. on the wall of the alveolus in the lung or the renal glomerula. The bi-layered arrangement, however, can be observed only during BM formation. Following the formation process and when the organ function starts, two layers are fused and seen as a uniform layer the thickness of which is less than twice the thickness of the L.d. [48,69,72].

**Fig. 7:**
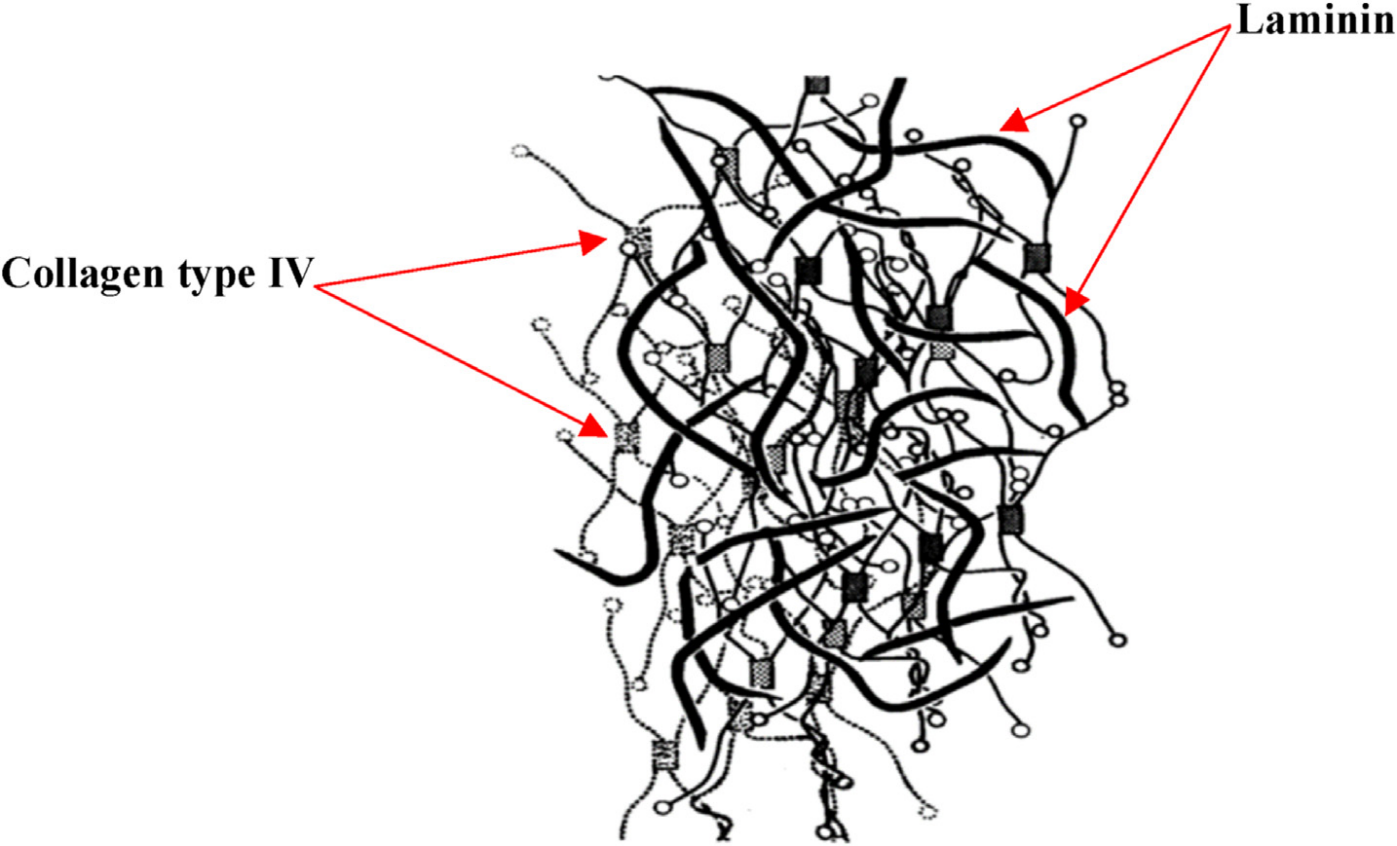
Schematic model depicting the structure of the Lamina densa, Thick lines represent laminin network and spider-shaped molecules are collagen type IV which are bonded at their globular ends [48]. Republished with permission of John Wiley & Sons Inc, from Morphology of the Basement Membrane, Merker, Hans-Joachim , Volume 28, Issue 2, 1994; permission conveyed through Copyright Clearance Center, Inc.

Morphological analysis has shown various complex structures within the L.d including cords, spatial spaces between cords and filaments, double tracks, basotubules, and double pegs. Cords are composed of irregular fluffy interlinked elements with an average thickness of 3.4 ​nm which form a meshwork [69,73]. With a diameter of 4.5–5 ​nm, the double tracks are present along with the cords. Two parallel running lines with an intermediary lighter band form the double tracks [74]. Basotubules are tube-like structures with a diameter of 7–10 ​nm. Finally, two parallel rodlets with 3.5 ​nm space in between form the double pegs [69,73]. The L.d is schematically represented in Fig. 7.

#### 3.1.2 Lamina lucida (L.l)

L.l extends from the plasma membrane of the cells to the L.d. It contains anchoring filaments with diameters of 2–8 ​nm which originate from the plasma membrane and attach to the L.d, tying the epithelium to the BM. Electron microscope images demonstrate a less dense and lucent layer compared to L.d (width of 15–65 ​nm) [48].

#### 3.1.3 Lamina fibroreticularis (L.f)

L.f is the most variable layer of the BM with regards to both composition and thickness. In fact, it is not present in embryonic tissues or in the double BM of the kidney, lung, around central-nervous-tissue capillaries, and in Reichert's membrane. It can, however, be as thick as 2 ​μm under the multi-layered epithelium. The thickest L.f can be found under the respiratory epithelium e.g. nasal mucosa and in the cornea [75,76]. This zone is a meshwork mainly composed of various collagens. It contains cords similar to the one in the L.d and thick ridges of L.d-like material extending from this layer into the connective tissues. Plaque-like structures (0.2–0.6 ​μm) can be observed underneath L.d, the composition of which is similar to that of L.d. Some anchoring filaments are composed of collagen type VII, referred to as anchor plaques radiating into the plaques. Collagen type III is the main constituent of the L.f, corresponding to ‘reticulin fibres’ or reticulum. In contrast to the fibrils constituting the lamina propia in the connective tissues, they do not form bundles and are not as thick as connective tissue fibrils. Fibronectin is another component of the L.f that can be observed underneath the L.d of the embryonic tissues, the function of which is important during the formation and stabilization of the BM. In mature tissues, however, it is responsible for the interlinkage of the structures in the L.f, for example anchoring plaques [77,78].

### 3.2 Chemical composition

The BM consists of more than 50 polymeric proteins such as collagens, elastin, laminins and fibronectin, which are fibrillar, and also glycosaminoglycans and proteoglycans. Collagens, generally, are fibrous structures with a triple-helical shape that are cross-linked allowing them to form higher-order structures. Collagens provide stiffness and flexibility. Glycosaminoglycans and proteoglycans, on the other hand, are in the form of hydrogels that offer resistance to compression and also provide reservoirs for growth factors [[79], [80], [81]].

The basal lamina is mainly comprised of collagen type IV, the glycoproteins laminin, nidogen (entactin), a heparan sulfate proteoglycan, and bullous pemphigoid antigen. The collagen type IV network is responsible for stabilizing and forming the layer structure. In fact, collagen type IV is a major component of the BM [82,83]. Other components of the BM are anchored in the collagen network. The entactin component binds laminin to collagen type IV. This binding capability is due to the integral component of the L.d. which is proteoglycan. The molecular arrangement of the components varies depending on the location of the BM [48].

### 3.3 Basement Membrane's functions

BM is a condensed polymer-like cluster of the ECM with many functions. Structurally, it provides a physical interface between the epithelium and the surrounding connective tissue as a demarcating layer. From a mechanical point of view, it plays a vital role in maintaining the integrity and stability of histological patterns. The great stability of the BM results in maintaining its structure even after the addition of various solutions and under ultrasonic treatment, making it valuable with regard to tissue regeneration. In the central nervous system of warm-blooded vertebrates, the regeneration of axons cannot occur partly due to the absence of BM around glial cells [84,85].

Another important function of the BM is its filtration feature. It is particularly of value in the glomerula of the kidney. The filtration properties of the BM are mainly due to the net-like structure of the L.d. layer [86,87]. Apart from the structural and physical functions of the BM, it can significantly influence cell behaviour. Morphogenetic functions of the BM are of paramount importance as it affects important processes such as differentiation, proliferation, migration, adhesion, axon growth, and cell polarization. These morphogenetic processes occur due to the linkage between the components of L.d. (laminin in particular) and the integrins of the cell membrane. Cell behaviour is controlled by BM's chemical composition. However, the physical and mechanical properties of the BM are also of great importance. Several studies have reported the effect of substrate stiffness on fundamental cellular processes such as attachment, growth, proliferation, migration, and differentiation [[88], [89], [90], [91]]. A study showed that hepatocytes responded better to soft plastic substrates than to solid ones. They tended to be round on soft substrates while a more flattened pattern was observed on solid substrates [48]. Another study reported the effects of collagen–glycosaminoglycan scaffold stiffness on differentiation and cell number. It was found that the scaffold with higher elasticity allowed increased cell-mediated contraction and led to a greater level of osteogenic maturation of MC3T3 cells. Lower levels of cell maturation and higher cell numbers, on the other hand, were seen using Stiffer scaffolds [92].

## 4 Key properties of the basement membranes

Gaining insight into cell-substrate interaction mechanisms is crucial for tissue engineering. BMs serving as substrates for overlying cellular structures regulate diverse cellular behaviour including adhesion, proliferation, migration, and differentiation. Biochemistry-dependent interactions have been studied extensively. Laminin, for example, can prevent cell migration, while hyaluronic acid inhibits cell-cell adhesion and promote cell migration [93,94]. The mechanism involves ligand-receptor interactions with the binding of cell membrane-associated integrin receptors to specific recognition sequences (such as arginine-glycine-aspartic acid, RGD) associated with the extracellular matrix being the most studied aspect [[95], [96], [97]]. However physical, mechanical, and topographical features [[98], [99], [100]] of the BM also significantly affect fundamental cellular behaviour. This highlights the significance of the identification of the physical, mechanical and topographical characteristics of the BM to improve the recapitulation of tissues. By developing substrates with surface features that correlate well with those found in the naturally occurring BM, appropriate cell function can be maintained. Moreover, these features are believed to affect the immune responses of tissue models which are often neglected despite their significant role in the healthy and disease states of every tissue. [101].

### 4.1 Mechanical properties of the basement membranes

Understanding the physical and mechanical properties of the BMs in detail is difficult due to their geometry, complex structure, tight junction to the cells and also the limitations of the existing analytical tools. Although it is crucial to identify the mechanical properties of the various BMs to control cell behaviour it is not possible to perform a precise analysis with current measurement methods and these properties have remained largely uncharacterized.

BMs show different mechanical properties, structures and thicknesses depending on their anatomical location. The composition and therefore the mechanical properties of the BM can vary in each tissue and even in different regions of the same tissue. Moreover, the characterization tool is another reason for the variability of the available data on the mechanical properties of BMs. Some isolation methods, for example, involved folding, stretching, and compression. transmission electron microscopy (TEM) characterization requires sample dehydration. This can lead to BM shrinkage due to the presence of proteoglycans which make up a substantial part of the BM [45]. Therefore, the deformation is different from what cells would experience *in vivo*. Although useful, the data for the mechanical properties of the BMs are approximate. Table 1 compares Young's modulus of different BMs. A large range in the reported mechanical properties of BMs from ∼kPa to ∼MPa can be seen due to stated reasons [45,[102], [103], [104], [105], [106], [107], [108], [109], [110], [111]].

**Table 1:** Comparison of Young's modulus of various BMs.

Tissue	Characterization Method	Young's Modulus (kPa)	Reference
Rabbit Renal tubules	Internal pressure	2000–5000	Welling et al. [105]
Rabbit Capillaries/Venules	Internal pressure	2000–5000	Welling et al. [105]
Rabbit Alveolar Capillary	Internal pressure	2000	Welling et al. [105]
Rabbit Alveolar Sheet	Internal pressure	3000	Welling et al. [105]
Inner limiting membrane (ILM)	AFM	950-3030 (chick)	Candiello et al. [45]
		3081-4070 (mouse)	
		1500-5000 (human)	
ILM	AFM	227 (retinal side)	Henrich et al. [112]
		44(vitreal side)	
The anterior BM of the human cornea	AFM	2–15	Last et al. [103]
Descemet's membrane	AFM	20–80	Last et al. [103]
Descemet's membrane	Volume-strain procedure	2810 (rat)	Danielsen et al. [113]
		6140 (cow)	
		4290 (sow)	
		2570 (human)	
Drosophila follicle	AFM	30–70	Crest et al. [106]
Drosophila malpighian tubule	AFM	∼1300	Howard et al. [108]
Drosophila Embryo ovarian follicule	AFM	20-800 (with development)	Chlasta et al. [114]
Mice renal tubule	Tensile^a^	Wild-type (WT): ∼438 and ∼3230 for low and high strain, respectively Peroxidasin KO: ∼284 and ∼2056 for low and high strain, respectively	Bhave et al. [110]
Mice mesentery	AFM	∼50	Glentis et al. [111]
Rat renal proximal and distal nephron	Microaspiration	o.5–1.5	Grantha et al. [115]
Breast cancer spheroids	Pressure-controlled inflation and deflation^b^	∼80	Li et al. [116]
Human posterior and anterior lens capsule	Tensile^c^	Posterior: 5400-55700	Krag et al. [117]
		Anterior:4400-44800	
Cat lens capsule	Tensile	820–7740	Fisher et al. [118]
Rat lens capsule	Tensile	650 (natural)-5100 (tanned)	Fisher et al. [119]
Human lens capsule	Tensile	2000–6000	Fisher et al. [120]
Lens capsule	Volume-strain procedure	540 (rat)	Danielsen et al. [113]
		1200 (cow)	
		1260 (sow)	
		2400 (human)	
Matrigel	AFM^d^ (biological conditions)	∼450	Soofi et al. [121]

a Force measurement cantilever was used in this study to which the isolated tubules were attached by applying a vacuum. The cantilever was connected to a manual micromanipulator while a holding pipette was connected to a motorized micromanipulator. The deflection of the cantilever and the distance traversed by the holding pipette were calculated using the acquired images at each deformation increment.

b The value represents the shear modulus and not Young's modulus.

c The test was performed by slipping the capsular rings over two pins which are connected to a motorized human anterior lens capsule micropositioner and a force transducer. The force and deformation were continuously recorded.

d The value is higher than the result for storage modulus previously reported in the literature (10–50 ​Pa). A parallel-plate rheometer was used in this study for characterization [122]. However, in a different report, storage modulus in the range of 20–300 ​Pa was reported for the Matrigel matrix depending on the concentration using a stress-controlled rheometer [123].

Values of elastic modulus of the BM in renal proximal and distal nephron were reported to be in the range of 0.5–1.5 ​kPa and were measured using microaspiration. According to Welling [105], the elastic modulus varies from 2 ​MPa for the BM of the alveolar sheet to 5 ​MPa for the BM of the renal tubules. Considering the differences in the thickness of these membranes, they showed remarkably similar stiffness. Nevertheless, the variation in the failure stress was larger (0.1–2 ​MPa).

The elastic modulus of the anterior BM and Descemet's membrane of the human cornea was measured using atomic force microscopy (AFM) [103]. Values were in the range of 2 ​kPa–15 kPa and 20 ​kPa–80 kPa for anterior and Descemet's BM, respectively. It is noted that these membranes are structurally similar. However, the difference in the stiffness of the two membranes can be attributed to the differences in the porosity and pore size differences seen in the structure of these membranes.

The results of AFM measurement for the BMs of chick and mouse internal limiting membrane (ILM) in a study showed that these BMs have greater thickness and, therefore, higher Young's modulus than what has been previously reported in the literature. They have a thickness of 402 ​nm and Young's modulus of 3.30 ​MPa and 4.07 ​MPa at embryonic day 9 for native chick and mouse, respectively [45].

As stated before only a limited range of BMs have been characterized for physical and mechanical properties with the lens capsule BM being the most extensively studied due to its easier separation from the lens cortex and is also the thickest BM in the body (approximately 5–10 ​μm for the anterior capsule and 20–30 ​μm for the posterior capsule in humans). Data shows that while the stiffness of lens capsules is almost 0.6 and 0.82 ​MPa for rat and cat, respectively, it is in the range of 0.3–2.4 ​MPa in humans [[117], [118], [119], [124]].

Another study reported the elastic modulus of the decellularized normal lung in the range of 1.6 ​± ​0.08 ​kPa using AFM as the method of mechanical characterization. It is not that different from the stiffness of the normal human lung which possessed a mean Young's modulus of 1.96 ​± ​0.13 ​kPa [125]. This suggests that the epithelial cells do not contribute significantly to the stiffness and the matrix is the main determinant. While this needs further investigation, and more study on the physical and mechanical characterisation of different BMs is certainly needed, it would be appropriate to consider the stiffness of tissue to be remodelled when there is a shortage of data on the mechanical properties of the respective BM.

Along with stiffness, other related mechanical aspects such as compliance and non-linear mechanics of the BM are crucial and should be considered in reproducing processes. The BM in the lung, for example, endures cyclic breathing-induced mechanical forces which cause stretching from 4% (normal breathing) at a frequency of 0.2 ​Hz (12–15 inhalation-exhalation cycles per minute) [[126], [127], [128]] to 12% (heavy exercises) at a frequency of 0.55 ​Hz (26–33 breathes per minute) [129]. It can even be stretched up to 20% in pathological conditions [130]. Lung BM-mimicking structures should be stretchable and robust to endure cyclic tensile forces without creep or fracture under biological conditions at least for the duration of the experiment.

One of the most recent studies on the elastic properties of the BM involved a pressure-controlled inflation and deflation test on the intact BM in breast cancer spheroids [116]. It was found that the BM exhibits a highly non-linear elasticity with a strong strain-stiffening effect. This non-linear stiffening behaviour is essential for avoiding the snap-through instability of the BM and therefore maintaining the integrity of tissues during growth owing to adequate confining stress [116]. This can be due to the presence of fibrous network structure in naturally occurring BMs. Non-linear elasticity is the typical behaviour of networks consisting of semi-flexible filamentous proteins [131]. The strain-stiffening effect in semiflexible fibrous networks can be due to the entropic effect of single fibres, filament bending or collective network effects which are governed by critical phenomena [132]. Matrigel (a commercially available BM complex), on the other hand, presents a wide linear elastic regime that indicates distinct structural differences which need to be addressed.

### 4.2 Topographic features of the basement membranes

Various microscopy analyses of BMs have demonstrated their distinguishing topographical features. Being the most studied feature of the BM, the thickness has been reported to vary from 50 ​nm to a few microns depending on anatomic location, age and characterization technique [45,103,133]. Our knowledge of the detailed topography of BMs,however, is still growing. Both characterised epithelial (corneal and Urothelial) and endothelial (Descemet and vascular) BMs of a few species have shown to have complex felt-like, 3d nanoscale topography which consists of intertwined fibres, pores, and elevations of varying sizes [[121], [122], [123], [133]]. The identification of substratum topography is crucial since it can specifically regulate cell behaviour including shape, orientation, adhesion, migration, and cell activation independent of ligand-receptor mediated pathways [[134], [135], [136], [137], [138], [139], [140], [141], [142]].

**Fig. 8:**
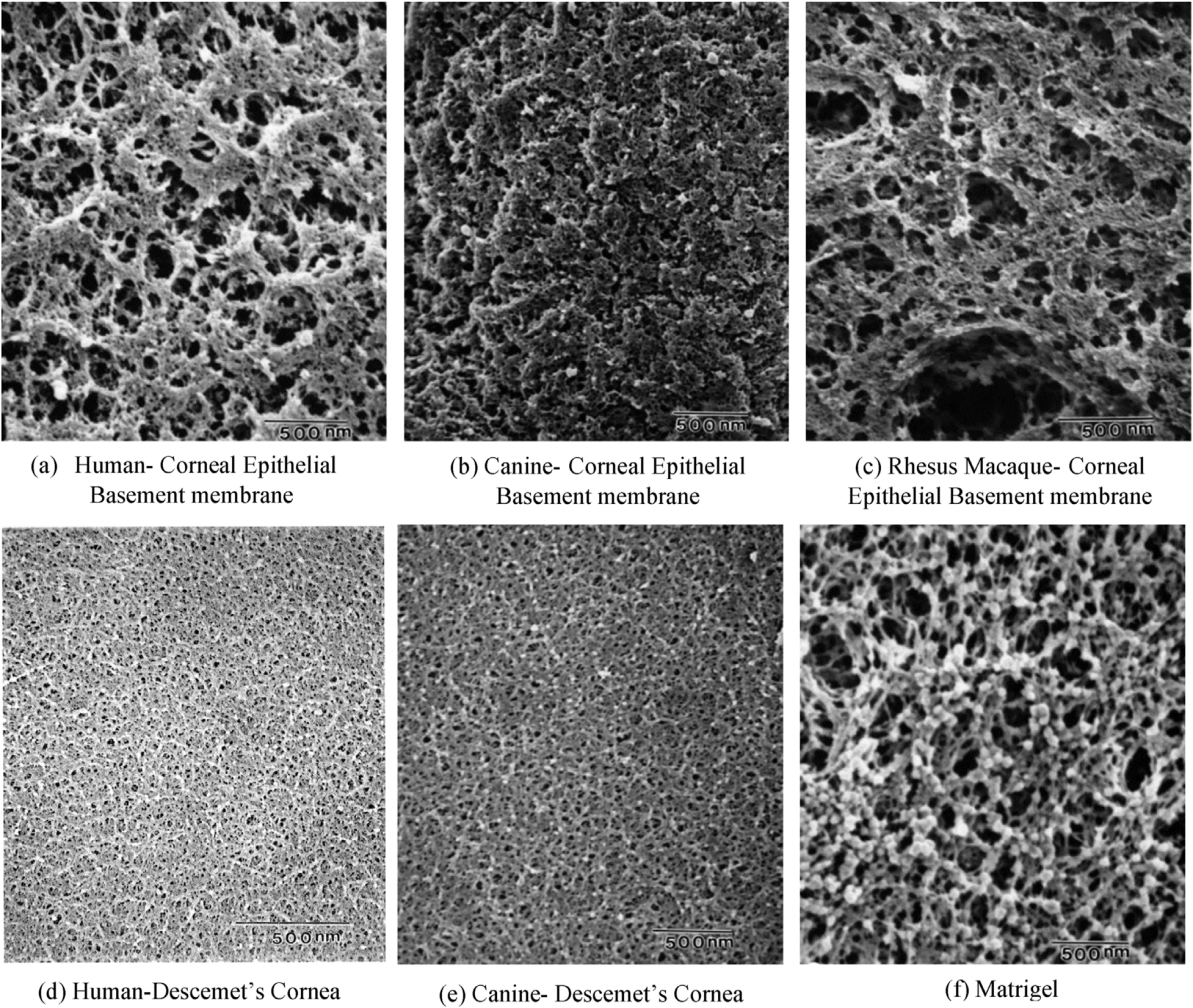
Scanning electron micrographs of the (a) Human [135], (b) canine [143], (c) Rhesus macaque corneal epithelial BMs [135] and (d) Human [144], (e) Canine Descemet's cornea [135], and (f) Matrigel, [135]. Images (a), (c), (e) and (f) republished with permission of Karger Publishers, from Electron microscopy of the canine corneal basement membranes, Abrams, George A., Ellison Bentley, Paul F. Nealey, and Christopher J. Murphy, Volume 170, 2002; Pages 251. Copyright © 2012 (or other relevant year) Karger Publishers, Basel, Switzerland. Image (b) reprinted from Investigative ophthalmology & visual science, Ellison Bentley, George A. Abrams, David Covitz, Cynthia S. Cook, Craig A. Fischer, Dennis Hacker, Charles M. Stuhr, Ted W. Reid, and Christopher J. Murphy, Microfabrication of human organs-on-chips, Vol. 42, Issue 10, Pages No. 2262-2269, Copyright (2001), with permission from Association for Research in Vision & Ophthalmology (ARVO). Image (d) reprinted from Cornea, Abrams, G.A., Schaus, S.S., Goodman, S.L., Nealey, P.F. and Murphy, C.J., Nanoscale topography of the corneal epithelial basement membrane and Descemet's membrane of the human, Vol. 19, Issue 1, Pages No. 57-64, Copyright (2000), with permission from AIP Publishing.

Fig. 8 demonstrate scanning electron micrographs of the epithelial and Descemet's membranes of a few species, as well as Matrigel. All BMs were found to have an intricate surface topography which is comprised of a heterogeneous mixture of fibres, pores, and elevations with the difference being in the size and geometry of the topographical features. The surface of the canine corneal epithelial BM, for example, is more porous and has bigger features compared to the endothelial surface of Descemet's membrane. While 18% of the surface of the epithelial BM was occupied by pores, they only comprised 10% of the surface of the Descemet's membrane [135].

Observations of various epithelial BMs revealed a consistent pattern of surface topography. Similar morphology can also be observed for different Descemet's membranes. Nevertheless, the feature sizes of Descemet's membranes are more similar to the same membrane in other species than they are to epithelial membranes in the same species. This could be due to the dynamics of the overlying epithelium and endothelium. While endothelial cells generally have no proliferation, epithelial cell turnover occurs every 1–2 weeks as a result of their exposure to harsher environmental conditions.

Table 2 compares the surface feature dimensions of various BMs obtained by various imaging techniques. Values obtained for elevations were similar for each species with human corneal epithelial BM elevation having the widest gap (the mean values obtained from SEM and AFM characterizations are 182 ​± ​49 and 243 ​± ​34 ​nm, respectively). While the Macaque corneal epithelial has the thickest fibres with a mean diameter of 77 ​± ​39 ​nm, the thinnest fibres were found in rat's Kidney Glomerular BM (5–10 ​nm) followed by Kidaortic heart valve ventricular with an average diameter of 28 ​± ​3 ​nm. With regards to pore size, the Matrigel showed the biggest pores (105 ​± ​70 ​nm) followed by human corneal BM (92 ​± ​34 ​nm). The smallest pores were, however, found in bovine's Kidney Glomerular BM (14 ​nm) followed by the aortic heart valve ventricular (28 ​± ​4 ​nm). Generally, epithelial BMs seem to have bigger topographical features compared to any other type of BM for all species with the exception of kidney tubular BM which seems to possess the smallest features among all investigated epithelial BMs. The BM in kidney demarcates the vasculature from the urinary space and is responsible for filtration. It enables the flow of plasma and small solutes while restricting the flow of larger plasma proteins. This can explain the small features reported for both tubular and Glomerular BMs [145,146]. More information on topographical features of the BMs can be found elsewhere [147].

**Table 2:** Comparison of dimensional values obtained using different imaging techniques for features observed in various BMs (SEM scanning electron microscopy, TEM transmission electron microscopy, AFM atomic force microscopy [103,[133], [134], [135]].

	Corneal BM (Epithelial)				Urothelial BM (Epithelial)	Descemet's membrane (Endothelial)				Kidney Glomerular BM (Endothelial)		Kidney Tubular BM (Epithelial)		Aortic heart valve (Endothelial)		Matrigel
	Human	Canine	Macaque	Matrigel	Macaque	Human	Canine	Macaque		Rat	Bovine	Rat	Bovine	Ventricular BM, porcine	Fibrosal BM porcine	
SEM																
Elevations																
Mean (nm)	182 ​± ​49	150 ​± ​41	190 ​± ​72	162 ​± ​52	178 ​± ​57	131 ​± ​41	115 ​± ​30	–	–	–	–	–	–	26 ​± ​13	22 ​± ​11	162 ​± ​52
Range (nm)	–	76–225	76–379	76–267	72–287	–	76–153	–	–	–	–	–	–	7–53	4–71	–
Pores																
Mean (nm)	92 ​± ​34	32 ​± ​18	71 ​± ​44	105 ​± ​70	82 ​± ​49	38 ​± ​15	24 ​± ​8	From 38 ​± ​2 for saphenous to 63 ​± ​6 for carotid		–	9	14	11	32 ​± ​20	28 ​± ​40	105 ​± ​70
Range (nm)	–	4–79	22–216	26–359	13–222	–	5–40	–		10-30, 9–14	–	–	–	12–75	7–98	–
Fibres																
Mean (nm)	46 ​± ​16	18 ​± ​9	77 ​± ​39	69 ​± ​35	52 ​± ​28	31 ​± ​9	15 ​± ​7	From 27 ​± ​1 for saphenous to 31 ​± ​1 for aorta		6	–	–	–	28 ​± ​30	30 ​± ​20	69 ​± ​35
Range (nm)	–	5–45	24–183	8–143	13–153	–	5–44	–		5–10	3–15	4–50	3–15	6–94	9–66	–
Interfeatured distance (nm)	–	40 ​± ​36	87 ​± ​36	117 ​± ​41	35–349	–	38 ​± ​15	–		8	–	–	–	–	–	–
TEM																
Elevations (nm)																
Mean (nm)	165 ​± ​78	119 ​± ​39	149 ​± ​60	–	–	107 ​± ​50	85 ​± ​21	–		–		–	–	–	–	–
Range (nm)	–	35–210	–	–	–	–	42–124	–		–		–	–	–	–	–
AFM																
Elevations																
Mean(nm)	243 ​± ​34	–	147 ​± ​42	196 ​± ​57	–	186 ​± ​45	–	–		–	–	–	–	–	–	196 ​± ​57
Range(nm)	–	–	79–371	24–386	–	–	–	–		–	–	–	–	–	–	–
Interfeatured distance(nm)	–	–	45 ​± ​23	22 ​± ​6	–	–	–	–		–	–	–	–	–	–	–

### 4.3 Limitations and challenges

Existing data on the physical and mechanical properties of various BMs in different tissues is very limited. Identifying the key properties of the BM is challenging due to their ultrathin nature, irregular shape and tight anchorage to cells. Isolation of this delicate structure from its adjacent interstitial connective tissue for characterisation is problematic. The limited suitable measurement techniques for the characterization of these sub-micron structures is another reason for the lack of data on the biophysical and mechanical properties of the BM [45]. Better isolation techniques or advanced characterization methods are highly required to address this issue. Developing a reliable constitutive model for the BMs can also be helpful to gain better knowledge of their physical and mechanical properties.

## 5 Membrane options and fabrication technologies

A variety of techniques have been used to fabricate porous constructs for OOCs, such as microfabrication techniques (soft lithography, replica moulding and phase separation micromoulding), rapid prototyping (RP)/solid free-form fabrication (SFF), Electrospinning and bioprinting. The applied technique and material choice considerably affect the main properties of the membrane including physical and mechanical properties.

Microfabrication employs techniques to fabricate micrometre-scale structures and is the commonly used approach to fabricate the micro-structured membranes for OOCs [30,148,149]. It is also used to fabricate the other parts of the chip. The porous flat membranes are mainly fabricated by two methods including soft lithography and track etching. The former is the most common method to fabricate both the rigid chip itself [148,[150], [151], [152]] and the membrane [64,148,152,153]. The process results in low pore tortuosity which is beneficial for the migration and transport of cells and nutrients. However, the process is not versatile and mainly uses elastomers like PDMS [154]. Commercial inserts or filter membranes made of poly(carbonate) (PC) and PET that are used as porous membranes for OOCs are often prepared by track etching [[155], [156], [157]]. This technique makes use of either electrons, heavy ions, X-ray irradiation, or UV light passing at predefined spaces of a mask. Although it results in low tortuosity cylindrical pores and offers great control over pore size, quantity and distribution, the use of chemicals or etchants is not preferable for most polymers [41].

Soft lithography replica moulding, phase separation micro moulding and thermoforming have also been used to fabricate porous microstructure membranes. Replica moulding is the most common soft lithography technique for chip fabrication [30,148,149,158,159]. However, it can also be used to create porous micro-structured membranes. Offering great control over the pores and microstructure, it can act as a controllable physiologically accurate representation of native tissue morphology. The process involves depositing the polymer on a microstructured mould before being cured, leaving negative copies of the microstructures of the mould after removal from the mould. The process seems simple. However, only one side of the membrane is left with microstructural features. Moreover, the position and orientation of the pores are limited. Soft lithography can be combined with particulate leaching to create pores. Pores, for example, were formed on PLGA scaffolds by dispersing glucose grains in the polymer solution and leaching them afterwards [160]. Phase separation micro-moulding is another technique capable of introducing pores and patterns to membranes in one step [154,[161], [162], [163]]. It involves phase separation of a polymer solution on a micro-patterned mould. Porosity can be tuned by the selection of solvent/non-solvent system and pore shape or size can be controlled by changing the design of the mould [[161], [162], [163], [164]]. It is a very simple, cost-effective and versatile method that can be used for a variety of polymers [154]. Moreover, intrinsic shrinkage during the phase separation facilitates the release of the replica from the mould [154]. However, it can be also a disadvantage as it can cause the deformation of the replicas of the features [165]. Thermoforming is based on a heated polymer sheet being drawn onto a mould with microstructure patterns by air pressure, vacuum, or mechanical forces. Polymer films can be track-etched before thermoforming to create a 3D structure. The wet-chemical etching is finally applied to open the pores [[166], [167], [168]]. Thermoforming has some limitations including not being versatile, uneven thickness in spots causing weak points, and is not suitable for achieving high aspect ratios [169,170].

Stated microfabrication techniques are mostly simple and cost-effective and are advantageous for the fabrication of microfluidics components. However, they are not suitable for the fabrication of an optimal membrane that can replicate the native BM. They lack the fibrillar architecture which is one of the main aspects of the BM *in vivo*. They also suffer from the lack of biochemical cues as processing BM proteins using these processing technologies is problematic.

Rapid prototyping (RP) which is also known as Solid Free-Form Fabrication (SFF) or additive manufacturing (AM) involves building 3D objects including scaffolds layer by layer in an additive manner from computer data such as Computer-Aided Design (CAD), computed tomography (CT) and Magnetic Resonance Imaging (MRI) data [171]. AM techniques enable the fabrication of constructs in desired geometry with high resolution and fully interconnected porous architecture. This benefit allows customization of scaffolds in shape, size and pore distribution. RP techniques for tissue engineering scaffolds fabrication include fused deposition modelling (FDM), selective laser sintering (SLS), stereolithography (STL), multiphoton polymerization (MPP)/two-photon polymerization (2 ​PP), and 3D printing (3DP) [172,173].

FDM involves extruding a polymeric fibre in the horizontal plane utilising a moving nozzle. Following the completion of the first layer, the plane is lowered and the process is repeated [[174], [175], [176]]. SLS utilizes a beam of infrared laser for sintering powder on a powder bed to build objects. Following the interaction of the laser beam and the powder, the local temperature increases to the glass transition temperature of the powder resulting in the infusion of the particles [177,178]. STL involves the polymerization of a liquid photo-curable monomer using an ultraviolet (UV) laser beam. The beam is directed onto the liquid surface by CAD data using a computer. The layers are scanned onto the surface of the resin. The first layers are attached to a platform which is then lowered for curing the successive layers [179,180]. Micro- or nanostructures can be prepared directly from a CAD model using MMP/2 ​PP technology which involves utilising tightly focused femtosecond-pulse-induced photo modification reaction in a confined volume [173,181].

Hydrogels are 3D cross-linked polymeric networks that retain a significant fraction of water within their structures. Either natural or synthetic polymers can be used to produce hydrogels. Synthetic polymers offer greater strength and are mainly hydrophobic and can be used to regulate the properties for specific applications. Hydrogels can be prepared using various techniques. Simply all techniques that are used to produce crosslinked polymers can be also used for hydrogel preparations.

However, photo-polymerization is usually utilized for producing hydrogels as it offers some advantages over traditional polymerization methods [182]. They include both spatial control over polymerization and pore formation, higher productivity (fast cure), lower reaction temperature and the ability to form complex shapes that adhere to the defect site [183,184]. The process involves utilising visible or UV light reacting with certain light-sensitive compounds to form hydrogel structures *in vitro*, *in vivo* or *in situ*. Although hydrogels are attractive materials for developing synthetic BM analogues, the limited mechanical stability of the hydrogels hinders their use in prolonged and dynamic cell cultures in microfluidics [185,186]. This can be addressed by using hydrogels in conjunction with fibre-processing techniques like electrospinning to develop a sufficiently robust hydrogel membrane reinforced with fibres [187].

Electrospinning is another spinning technique and it is a powerful method to fabricate fibrous membranes that can closely mimic the native ECM in a simple and low-cost way [[188], [189], [190], [191]]. The electrospinning technique is capable of producing continuous fibres with diameters ranging from microns to nanometres in size. These micro/nanofibrous structures simulate ECM architecturally and also scale-wise [192]. These properties combined with high porosity, surface area to volume ratios, pores interconnectivity, and the simple and versatile nature of the process makes electrospinning promising to replicate the natural ECM [188,189]. Fibre diameter, porosity and the mechanical properties of the constructs can be controlled by the electrospinning parameters including extrusion rate, solution concentration, applied voltage, the distance between the nozzle and the collector and the collector unit. Moreover, electrospinning is conducted at room temperature allowing for using temperature-sensitive polymers as well as bioactive agents [193,194]. Another interesting characteristic of nanofibres is their dynamic, or deformable, pores. Randomly oriented fibres are deposited loosely on one another to create the construct. The pathways for cell infiltration can be expanded as cells migrate through flexible but mechanically strong fibres. This explains cell infiltration through the nanofibrous membrane with a smaller diameter than the pore dimension [188]. It is assumed that cell freedom to adjust pore diameter comes from the dynamic scaffold architecture. Electrospinning is a very suitable technique for the fabrication of artificial BMs since they offer fibrillary structures with high porosity and can be used to process natural proteins. One limitation of electrospinning can be reproducibility due to the chaotic nature of the process; Nonetheless, it can be minimised by careful optimization of the process. The toxicity of the residual solvent can be another issue that should be addressed before cell culture [195]. Cell infiltration can also be problematic due to the close packing of nanofibers [194,196,197]. This can be addressed by processes like multimodal electrospinning where nano- and microfibre populations can be formed simultaneously [198].

3D Bioprinting is a method based upon depositing bioinks through a nozzle with either encapsulating cells or loading cells within the bioink. This technique allows printing the bioink in the required shape in order to form subtle structures that can be compared to tissues [199]. 3D bioprinters usually consist of either a moving nozzle with a fixed platform or a fixed nozzle with a moving platform in three dimensions (x, y, z-axis). The nozzle's movement is controlled by the coordinates acquired from the CAD file [200]. Over the past years, 3D bioprinting applications have been massively increasing due to merits such as cost-effectiveness, simplicity, relatively precise deposition and cell distribution controllability [201]. In order to enhance the printability parameter of 3D bioprinters, bioinks should be furtherly investigated and optimized for a larger extent of applications.

Bioinks possess a substantial amount of significance considering they are the main constituent of the 3D bioprinting concept. Recently, there are various studies in the literature regarding bioink development and printability [202]. The majority of the studies concern synthetic and natural polymers including in the form of hydrogels. Some of the materials used as bioinks and their properties are noted in Table 3. The endless potential of creating new bioinks for better biocompatibility, precise resolution, and biomimicry lies ahead to overcome the limitations.

**Table 3:** Comparison of bioinks currently used in TE applications [203].

Materials	Advantages	Disadvantages	Crosslinking methods
Alginate	Relatively low cost, high gelation rate, biocompatible, good printability	Relatively low cell adhesion, can degrade during culturing	Ionic
Agarose	simple, mediocre mechanical properties, good stability	Poor cell adhesion, not biodegradable	Thermal
Methylcellulose	Decent printability and biocompatibility	Can degrade during cell culture, not ideal for long-term culturing	Thermal
Chitosan	Biocompatible, can be processed to be antibacterial	Poor cell adhesion, slow gelation rate	Ionic or covalent
Hyaluronic acid	Substantial cell proliferation, high gelation w/modifications	Fast degradation, low stability	Ionic or covalent
Gelatin	Relatively cheap, High cell adhesion and viability, biocompatible	Low printability, poor mechanical strength	Thermal
GelMA	Versatile, good biocompatibility, tunable properties, photocurable	Relatively low printability	Photocurable, UV light
Polyethylene Glycol Diacrylate (PEGDA)	Photocurable, relatively biocompatible	Requires chemical modification, not degradable	Photocurable, UV light
Collagen	High cell adhesion and viability, high biocompatibility, self-assembly	Low viscosity, Blending and/or cross-linking is required for printability	Thermal or ionic
Decellularized extracellular matrix	High cell adhesion and viability, tunable for specific tissue applications	Requires intensive processing for preparation, may contain immunogens	Ionic or covalent

3D-bioprinting can be used to fabricate microfluidic devices from hydrogels [204,205] or to directly print hydrogels into a pre-fabricated chip [206,207]. Low resolution is a major drawback of 3D-bioprinting. This results from the flexibility of the bioinks and long curing during which the homogeneity of cell distribution can be affected [208]. Light-assisted bioprinters have been used to address this. However, they are not versatile concerning the material choice and they can also cause cytotoxicity [209]. The applied high shear stress to cells during extrusion is another challenge and it is even more problematic when a smaller orifice is used to improve the resolution [210].

## 6 Polymers used in the preparation of membranes for OOCs

Although a wide variety of natural and biocompatible polymers have been used in the fabrication of scaffolds only a handful of polymers have been used to fabricate the porous membranes for the OOCs application with PDMS, PC, PET, Polylactic acid (PLA) and poly(1-caprolactone) (PCL) being the most studied. This is mainly due to practical reasons and to minimise the complexity of the system to avoid unpredicted effects. Biodegradability of the polymer, for example, could change the properties of the membrane over time and may cause cytotoxicity. PDMS is the most widely used polymer for the fabrication of the porous membrane and also the other parts of the microfluidic chip. This is due to its easy production process, flexible nature and transparency which is desirable for drug studies [30,64,148,149]. With an elastic modulus of a few MPa, this polymer is most suited for stretch devices i.e. *in vitro* lung or skin models. However, this polymer is highly hydrophobic and needs to be modified using surface modification approaches to improve its cell affinity [211,212]. PC is also a common polymer to fabricate porous membranes for OOCs [156,213,214]. PC is highly hydrophobic and stiff with Young's modulus around 2–2.4 ​GPa which is not comparable to the stiffness of the BMs. However, it is often used because of its wide application and acceptance in Transwell cell culture inserts. PC membrane surfaces need to be coated with proteins or functionalized with surface modification techniques to improve cell attachment [215]. PET with Tg of 70 ​°C is a relatively stiff polymer (Young's modulus of 2–3 ​GPa) and is not suitable for stretch devices. It is less hydrophobic than PDMS and PC (water contact angle of 82.6°) and is also transparent [216]. PLA is an aliphatic polyester and with Young's modulus of 3–4 ​GPa is one of the stiffest biocompatible polymers. It is, however, less hydrophobic compared to other polymers being investigated for OOCs (Water contact angle of 61°) [159]. PCL is another aliphatic polyester and with water contact angle of 119° is one of the most hydrophobic polymers. Its glass transition temperature (Tg) of −60 ​°C results in the material possessing a rubber like consistency at room temperature which makes it a suitable material for stretch *in vitro* models [217]. However, PLA and PCL are both biodegradable and this needs to be taken into consideration in the design of the membrane.

Table 4 shows various OOCs consisting of different membranes in terms of material selection and fabrication techniques. Microfabrication techniques such as soft lithography and track etching, electrospinning and bioprinting seem to be the most widely used methods for membrane fabrication used in the microfluidic systems. Although the presented OOCs have advantages as detailed in the Table 4, there are still some limitations regarding the utilized membranes which need to be addressed for the development of more physiologically relevant tissue models. For example, microfabricated membranes from non-biodegradable polymers used in the presented OOCs to replicate various organs such as lung and gut are too thick to be a representative of the native ultrathin BM. Thick membranes pose a challenge regarding the transport of the oxygen, protein, biomolecules, and immune cell across the barrier [218,219]. Moreover, with a smooth surface with artificial pores, they poorly replicate the fibrillar structure of the *in vivo* BM. This can alter the topographical cues thus changing the original cell phenotype [133]. Irreversible permanent bonding between the layers of the microfluidic device is another limitation as it makes some characterizations such as histology and electron microscopy challenging. Moreover, in case of any part failure, the whole device must be discarded. They also possess a high elastic modulus (with PDMS being an exception) in the GPa range which is significantly stiffer than the native BM. Electrospun membranes represent the native BM better regarding architecture and provide more relevant topographical cues for cells. However, the reported average fibre diameter is still hundred times larger than those in the BM (<100 ​nm, see Table 2). Moreover, they are still stiffer than the natural BM with Young's modulus in the hundreds of MPa range. Hydrogels have also been incorporated in the microfluidic devices using different techniques. Although they provide relevant biochemical cues they are sometimes not sufficiently robust for enduring mechanical forces. Their biodegradability can also be another challenge for microfluidic applications. They also lack the fibrillar BM structure.

**Table 4:** Overview of OOCs utilising membranes developed from different technologies that employed *in vitro* studies on cellular behaviour.

Material	Manufacturing method	Device	Cell type/Cell culture experiment duration	Thickness (μm)	Pore size (μm)	Modulus	Important remarks	Ref
PDMS coated with fibronectin	Microfabrication- Soft lithography	Lung & Gut-on-chip	Human alveolar epithelial cells, microvascular endothelial cells-human intestinal epithelial cells (Caco-2)/20 days	10	10	–	Successful formation of confluent monolayers of human lung epithelial cells and pulmonary microvascular endothelial cells on the opposite sides of the porous flexible PDMS membrane proved the applicability of the developed alveolar-capillary interface. 3D villus-like structures were also successfully formed in the Gut model.	[148]
PDMS coated by type I Collagen and Matrigel	Microfabrication- Soft lithography	Gut-on-chip	Caco-2/Transwell: 21 days, Microfluidic device: 5 days	30	10	–	Caco-2 ​cells were cultured on a stretchable PDMS membrane where they were under low shear stress (0.02 ​dyne ​cm^−2^ and a cyclic strain (10%; 0.15 ​Hz) from the side chambers.	[30]
PDMS coated by type I Collage & Matrigel	Microfabrication- Soft lithography	Gut-on-chip	Caco-2/21 days		10		Villi-like gut epithelium was developed onto a porous flexible PDMS membrane within a microfluidic chip. Cultured human Caco-2 intestinal epithelial cells were exposed to physiological peristalsis-like cyclic mechanical strains and fluidic flow.	[149]
PDMS coated by human fibronectin or gelatin/collagen I	Microfabrication- Soft lithography	Lung-on-chip	Bronchial epithelial 16HBE14o-Primary human pulmonary alveolar epithelial cells (pHPAEC)/21 days	3.5 or 10	3 or 8	–	16HBE14° and pHPAEC were cultured on the apical and basal sides of the porous stretchable membrane, respectively. It was found that the cyclic mechanical strain significantly affected the permeability properties of the cells.	[64]
PLLA	Microfabrication- Soft lithography	A microfluidic chip	Human umbilical vein endothelial cells (HUVECs)/7 days	∼100 ​nm	2	∼2.3 ​GPa	Ultrathin PLLA membranes with patterned micrometric pores were fabricated by spin coating-assisted deposition of the polymer solution on polyvinyl-alcohol (PVA) replicas (a sacrificial array of spatially ordered polyvinyl-alcohol nanoneedles). HUVECS cells were then cultured on top of the integrated membrane in a microfluidic chip to test the suitability of the permeable membrane.	[159]
PC	Microfabrication-Track etching	Brain-on-chip	Primary human brain-derived microvascular endothelial cells (hBMVEC)- astrocytes/12 days	0.2	–	–	The blood-brain barrier was replicated using a microfluidic device with two chambers separated by a commercially available 0.2-μm polycarbonate membrane to study the BBB response to immune activation.	[155]
PC	Microfabrication-Track etching	Liver-Intestine, Liver-skin	HDMEC human dermal microvascular endothelial cells, HHSteC human hepatic stellate cells, RBC human red blood cells/14 days	10	0.4	–	A multi-organ-on-chip platform was developed for the first time for the co-culture of human 3D liver spheroids either with human intestinal epithelial cells or skin biopsies. A 14-day co-culture confirmed the capability of the system in maintaining different human organ equivalents.	[220]
PET coated with collagen type IV	Microfabrication-Track etching	Human kidney proximal tubule-on-chip	Primary human kidney proximal tubular epithelial cells/6 days	10	0.4	–	A collagen type IV pre-coated PET membrane that was developed by ion-track etching was used to separate microfluidic channels in a chip. Primary kidney epithelial cells were cultured on the membrane under apical fluid shear stress to recreate the human proximal tubule microenvironment for assessing renal physiology, kidney diseases, and nephrotoxicities.	[158]
PET	Microfabrication-Track etching	WAT (white adipose tissue)-on-chip	3T3-L1 fibroblasts/9 days	20	3	–	The physiological environment of adipose tissue was replicated by developing a microfluidic chip composed of salinized microporous PET membrane which was sandwiched between a media channel and circular cell chambers. The system featured functional lipid metabolism for more than two weeks.	[221]
PLLA	Electrospinning	A microfluidic system	MC3T3-E1 preosteoblast cell line/3 days	∼30, ∼100	–	∼ 2.6- ∼ 3.9 ​MPa	This microfluidic device was developed on aligned and randomly oriented electrospun PLLA membranes as a lateral-flow model for cell culture- Liquid flow was possible due to capillary actions resulting from the fibrous nature of the membranes.	[222]
PCL	Electrospinning	A microfluidic chip	Primary human dermal fibroblasts, RAW 264.7 macrophages/3 days	379 ​± ​15	113 ​± ​19	–	PCL fibres were electrospun directly into a fully sealed fluidic channel using dynamic focusing electrospinning. The coating on the inner side of a fluidic channel with PCL fibres enhanced the production of cytokines such as interleukin-6 (IL-6) and vascular endothelial growth factor (VEGF).	[151]
Silk fibroin- PS- PCL	Electrospinning	A microfluidic chip	Bovine pulmonary artery endothelial cells-macrophages (RAW 264.7)/Endothelial cells: 24 ​h statice culture. They were then transferred to the microfluidic device for a further 24 h- Macrophages: 24 ​h.	100	12.8 ​± ​2	–	Fibrous inserts were prepared and were modularly integrated into the microfluidic chip. Macrophages cultured on fibrous silk fibroin membrane showed more physiologically relevant response rates than those cultured on a flat surface under lipopolysaccharide (LPS) stimulation.	[223]
4% PEG- 96% PCL coated with type I collagen	Electrospinning	The blood-brain barrier (BBB)	Human-derived endothelial cells, pericytes and astrocytes/7 days	∼6	∼0.6	–	The Human BBB model was developed using electrospun BM-like PCL and PCL-PEG (96-4%) substrates coated with collagen in a chip. Human-derived endothelial cells, pericytes and astrocytes were cultured on the membranes under static conditions.	[217]
PLA- GelMA	Electrospinning	Thermoplastic-based organ-on-chip	Human (microvasculature) endothelial -(retinal pigment) epithelial cell layers/7 days	–	∼0.6	5 or 1	Membranes were fabricated by direct electrospinning 12 ​wt% PLA and PLA: GM2) solutions in HFIP onto the PMMA. The good viability of mvECs and RPEs cells in the organ-on-chip system proved the suitability of the system as a more biomimetic *in vitro* cell culture system.	[224]
PCL	Electrospinning	A microfluidic chip	Human hepatic carcinoma cells (HepG2)/14 days	113.7 ​± ​2.7	0.3 and 100	–	100 ​μm thick membranes with a porosity of 76% were fabricated by electrospinning 8 ​wt% concentrated PCL-Chloroform solutions and were integrated into a microfluidic chip. HepG2 proliferated twice as rapidly under perfused conditions as did under static conditions.	[225]
Polyurethane (PU)	Electrospinning	A microfluidic chip	human mesenchymal stem cells (hMSC)/5 days	35	5–10	–	The electrospun PU membranes were plasma treated to improve their hydrophilic characteristics. The result revealed that plasma treatment and flow rates in the microchannels significantly affected cell proliferation.	[226]
PVDF	Electrospinning	A microfluidic chip	–	30–50	–	–	Electrospun PVDF membranes were integrated into a microfluidic chip using a scotch tape-assisted method for multiple immunoassays. Protein adsorption was found to be eight times higher on electrospun membranes than on commercial track-etched polycarbonate membranes.	[227]
PLGA	Electrospinning	Lung-on-chip	Human non-small cell lung cancer cells (A549) and human fetal lung fibroblasts (HFL1), HUVECs/5 days	0.3 and 0.7	–	–	A549 and HFL1 cells were cultured on top and bottom of the electrospun PLGA membrane in one microchannel of the chip, while HUVEC cells were cultured on the membrane in the side microchannel. A549 cells caused HUVEC cell apoptosis or death which can result in tumour cell invasion.	[228]
Nylone 6 and Nylone 6-collagen-PLLA	Electrospinning	Lab-on-brane	HSMCs, HAEC/48 ​h	70 ​± ​20	0.28–10.7	Nylon-6: ∼ 405 ​kPa Collgen/PLLA:∼515 ​kPa	Nylon and Collagen-PLLA [1] solutions were directly electrospun onto the PDMS chambers and also on patterned substrates and manually put over the PDMS chambers. HSMCs and HAEC cells were then co-cultured on the membrane to accurately simulate arterial anatomy.	[229]
GelMA	Infiltrating & cross-linking of GelMA into the void spaces of a pre-made lattice of alginate microbeads followed by the selective removal of microbeads.	Alveolar-on-chip	Primary human alveolar epithelial cells (hAECs)/14 days	∼3 ​mm	325.9 ​± ​10.5, 199.6 ​± ​1.1, 156.3 ​± ​2.7	∼ 6.23 ​kPa (compressive modulus)	Capturing some of the main aspects of the alveoli, structurally, also possessing ALI and relevant ECM microenvironment, the system showed superior maintaining of the functions of hAECs compared to planar models.	[37]
Collagen	Filling uncured hydrogels in the central channel of a microfluidic device and then crosslinking process.	Multiwell capillarity-based microfluidic device	HUVECs, MDA-MB231 tumour cells	–	–	–	A mixture of collagen and the cancer cells suspension [1] was used to fill the plasma-treated central cannel and then polymerized. This microfluidic chip was designed to investigate the penetration of TNF-related apoptosis inducing ligand (TRAIL) into the endothelium to destroy tumour cells in a 3D collagen matrix.	[230]
Collagen	The injection of hydrogel into the microfluidic chip following by crosslinking process.	Blood-brain barrier	Primary rat astrocytes, neurons, human cerebral microvascular endothelial cells (hCMEC/D3), HUVEC/hCMEC/D3: 7 days, HUVEC: 4 days	–	–	–	A mixture of Collagen solutions, astrocytes suspension and neurons were injected into a chip and was then polymerized. This neurovascular chip was designed to study the effect of drugs on neurocytes and astrocytes.	[231]
Collagen	3D photopatterning technology (using a photomask to form hydrogel pillars)	Skeletal muscle-on-chip	The C2C12 mouse murine myoblast cell line/12 days	–	–	–	This technique involves fabricating two hydrogel pillars within the microfluidic channel and then the polymerization of the cell-laden hydrogel solution in a capsule shape between the pillars. This process is advantageous compared to the direct injection of the hydrogel solution into the microfluidic channel because it enables spatially organization of the cells within a hydrogel network around the anchoring pillars.	[29]
Hyaluronic acid (HA) and gelatin	3D photopatterning technology (using a photomask to form islets of cell-laden hydrogels within flow paths	Liver-on-chip	HEPG2/7 days	–	–	–	The hydrogel was mixed with a solution derived directly from the liver ECM for a better resemblance to the native tissue. The innovative design of this microfluidic system enabled better molecular diffusion as the media could flow around the islets which resulted in functional cells over 7days.	[232]
Alginate, GelMA	Bioprinting	Heart-on-chip	HUVEC, human iPSC (hiPSC)-derived cardiomyocytes/HUVEC 33 days, cardiomyocytes were seeded on day 15.	–	∼2.44, 7.33	–	Endothelial cells encapsulated microfibrous lattices were developed using bioprinting. They were seeded with cardiomyocytes to form a myocardium capable of spontaneous and synchronous contraction. They were then combined with a microfluidic device to form the endothelialised heart-on-a-chip device for studying drug effects.	[233]
Cell-laden Matrigel [1]	Bioprinting	Liver-on-chip	HEPG2, human mammary epithelial of the cell line M10/48 ​h	–	–	–	Cell-laden Matrigel was printed directly onto a PDMS substrate using bioprinting. The bioprinted constructs were sealed in microfluidic chips which were connected serially to create dual-micro-tissue microfluidic chips. Multi-cellular drug conversion and effectiveness of the radiation shielding by pharmaceutical amifostine were studied using this system.	[234]
HepG2/C3A hepatic spheroid-laden GelMA	Bioprinting	Liver-on-chip	HEPG2/C3A hepatic spheroid/30 days	–	–	–	HepG2/C3A hepatic spheroid-laden solutions were printed on a glass slide within a cell culture chamber and then cross-linked by immediate UV light illumination to form hydrogel constructs. Long-term culture of 3D human HepG2/C3A was possible using this system to assess drug toxicity.	[206]

Most of the studied OOCs use cell lines that are not relevant to *in vivo* cells. Due to the physiological and metabolic differences between animals and humans, it will be necessary to replace animal cells with human cells in future OOCs designs [4]. Human-derived cancer cell lines are also being abundantly used in OOCs. Nevertheless, the use of primary human cells should be considered as cancer cell lines poorly replicate the phenotype of original cells [64]. Using only one or two cell types is another limitation with most current OOCs. The lack of other components of the organ which have a significant role in organ homeostasis and pathogenesis should be addressed to better recapitulate relevant tissues [148].

## 7 Optimal artificial basement membrane

The recapitulation of the multi-component native BM cues *in vitro* is challenging and tailoring the biochemical, biophysical, topographical and mechanical properties of the BMs is intricate. While some properties of the BM are tissue-specific such as thickness, permeability, and stiffness, they have common characteristics; They are mainly composed of similar proteins with defined structural building blocks of nano-micro fibres.

Silicone-based membranes such as PDMS developed using biofabrication technique are often used as a representative of the BM in cell-stretching devices either in static cell culture conditions or microfluidics. They provide several advantages such as optical transparency for cell observation, stretchability, and facile integration with the microfluidic chip [41,235]. However, they suffer from poor replication of the BM nano fibrillar architecture, high hydrophobicity, lack of biochemical cues, and the unwanted absorbance of some drugs and biomolecules [218,236]. PC and PET membranes have also been abundantly used for similar applications. Although they have shown less absorbance of drugs high stiffness of these materials can be a great challenge for BM-mimicking structures [235,237]. Another limitation with these membranes is their thickness (∼10 ​μm), decreasing permeability of the membrane which is essential for active molecular communications and functions of the cells [238].

BM proteins such as collagen IV, laminin and fibronectin, on the other hand, introduce desirable cell affinity which facilitates a fully confluent cell monolayer on the developed membranes [148,239]. These proteins are extracted from biological tissues and then processed into a hydrogel form which can represent the BM [240,241]. Although providing the required biochemical cues for cells and being highly porous, they are not sufficiently robust to form a thin membrane for integration into microfluidic devices or even to endure prolonged mechanical forces during cell culture which requires at least 1 week for cell maturation [187]. Moreover, meeting the stretchibility requirement to mimic the cyclic mechanical strain *in vivo* is challenging. Surface modification of membranes made from synthetic polymers (i.e coating with BM proteins or plasma treatment can introduce surface cell recognition sites or recude the hydrophobicity and thus improve cell attachment while addressing the mechanical aspect requirement [64,158,221]. Nonetheless, the bulk material is still unable to mimic the biochemical composition of the BM and therefore hinders the process of physiological cellular phenotypes expression due to a lack of instructive cues.

Along with the stated considerations, technical aspects of using BM-mimicking materials within OOCs should also be taken into account. The integration of a free-standing ultrathin membrane with low stiffness into microfluidic devices that can remain intact and flat across the channel width during and after assembly is a dilemma. Optical transparency is also an important aspect that needs to be considered for direct observation and analysis of cells which is often lacking in membranes such as electrospun nanofibers and Transwell inserts. Potential degradation of synthetic BM structures under flow conditions is another challenge. This occurs due to erosion or enzymatic degradation resulting from the presence of the enzymes in the culture medium (via commonly used serum). Use of defined media is a potential solution for the latter deterioration and for the former crosslinking of the structures provide more resistance to both flow and flow-induced shear stress conditions. However, such crosslinks (particularly if obtained through chemical or photocrosslinking routes) can significantly change the physicochemical properties of the BM components together with their biochemical activity. Thus, such modifications should be rather done through enzymatic crosslinking or via supramolecular interactions for better mimicking the ECM microenvironment.

Currently, there is no widely accepted material or method that can meet all the requirements for the BM. Designing an artificial membrane that can provide biochemical, biophysical, topographical and mechanical cues while can also address technical issues within microfluidic devices is highly necessary.

## 8 Incorporation of more biomimetic membranes in OOCs

The integration of scaffolds into microfluidic chips has been recently explored to develop more biomimetic tissue models. Applying the scaffolding materials in the chamber of the chip depends on the type of scaffold. Hydrogels and electrospun membranes seem to have the closest morphology to the native BM and have been the most studied materials to improve the physiological relevance of the system.

OOCs devices require micron resolution and therefore the incorporation of the hydrogels in these microfluidic devices should be based on filling the pre-made hollow channels with curable hydrogels or existing channels from hydrogel bulk materials. The former commonly involves injecting cell-laden hydrogels to fill the channel through capillary action followed by curing. The media then is perfused through the side channel to provide nutrients and oxygen to cells through diffusion [230,242,243]. Although simple, these methods lack efficiency with cells experiencing necrosis in a gel thicker than 200 ​μm due to insufficient supplies of oxygen and nutrients [238]. In a study, a blood-brain barrier was modelled by injecting cell-laden collagen solutions into the plasma-treated central channel of a chip and then was polymerized for 30 ​min at 37 ​°C [231]. To improve molecular diffusion, islets of cell-laden hydrogels within flow paths were fabricated using a photomask by Skardal et al. This technology allows for the precise localization and patterning of cells [232]. Apart from the direct injection of cell-laden hydrogel solutions into the microfluidic channels, microfluidic chips can be developed from hydrogels using either replica moulding or 3D bioprinting. This can address the issue of photocuring, however, hydrogel microfluidic devices are not mechanically strong [242,244]. Along with the fabrication of bioprinted microfluidic devices from hydrogels, bioprinting of biological construct can be also done directly into a microfluidic chip. Bhise N. et al. made use of bioprinting encapsulated hepatic spheroids into a microfluidic device to develop a liver-on-chip [206]. Daniela et al. explored the feasibility of an engineered 3D *in vitro* model with on-chip vascularized channels using directly bioprinting elastin-like protein engineered hydrogel onto an already endothelialised on-chip platforms [207].

Researchers have used different methods to integrate electrospun fibrous materials into microfluidics. Methods include the fabrication of the microfluidic device from electrospun sheets, direct electrospinning of fibres into the microfluidic channel or peeling off the membrane from regular collectors and manually transferring it into the microfluidics followed by typical sealing methodologies, and modular integration of nanofibers in microfluidic channels.

A microfluidic device was developed on a sheet of poly(l-lactic acid) electrospun fibres by blocking certain areas to form hydrophobic barriers and leaving the areas for the formation of the channels and circular zones untreated [245]. Although simple and low-cost the use of cells requiring shear stress within this system would be problematic.

Direct electrospinning of fibres into microfluidic channels can be done by modifying the electrospinning setup to guide fibres to the channels of the chip. This was done by incorporating a 3D printed sheath device around the nozzle for direct focusing of PCL fibres into a fully closed fluidic device [151]. The secretion of cytokines from the cultured macrophages on the fibre scaffold was improved using this system. Although the formation of the electrospun fibres on the channel wall was confirmed by SEM imaging, developing a highly uniform layer of nanofibers can be challenging. Focusing fibres toward a specific target is difficult as fibres tend to spread out during electrospinning. Moreover, very small microfluidic channels cannot be used as target collectors in this setup which is another limitation.

Karim et al. [229] used a different integration approach for developing a blood-vessel lab-on-a-brane system to recreate *in vivo* vessel-tissue interface for assessing transendothelial transport. Electrospun-coated PDMS chambers as well as their corresponding sandwich counterpart chambers were then plasma-treated, clamped and cured at 70 ​°C for 90 ​min to ensure a secure bonding of the sandwiched membrane in a watertight seal. The nanofibrous membrane in conjunction with adsorbed attachment factors proved to be an effective ECM for the co-culture of smooth muscle cells and endothelial cells.

Another study took advantage of the integration of PU nanofibre scaffolds into the micro-channels of a microfluidic chip to study the function of hMSC. Fibres were spun on an aluminium foil and then peeled off, treated with plasma for acrylic acid (AA) grafting, and manually transferred onto the oxygen plasma-treated lower layer of the chip (microfluidic channel). It was then bonded to the plasma-treated upper layer (cell culture chamber) and cured at 80 ​°C for 2 ​h under pressing forces (∼0.89 ​kg/cm^2^). A greater hMSC adhesion, migration, and proliferation were observed for the AA-grafted PU surface as compared to the untreated one using this microfluidic cell chip. [226].

PLGA nanofibre membrane supported lung-on-chip microdevice was developed by Xingyuan Yang for anti-cancer drug testing [228]. Similar to the previous study, nanofibres were directly spun on the PDMS upper layer of a microfluidic device. The chip was then placed on the middle hole of a confocal dish. With nanofibres providing a 3D cell culture environment, the co-culture of three kinds of cells (A549, HFL1, and HUVEC) was possible. It was found that A549 ​cells showed resistance against gefitinib, an epidermal growth factor receptor (EGFR)-targeted anti-tumour drug. This can be due to the IGF-1 secretion from HFL1 cells that maintain the tumour cells by preventing the EGFR-related signal pathway by the drug and activating the PI3K/Akt signal pathway.

Yingyi Liu et al. [227] developed a microfluidic chip with electrospun polyvinylidene fluoride (PVDF) as the membrane. The integration method involved placing a piece of scotch tape on the PDMS slab with an adhesive surface facing upward on which the already spun fibres on the aluminium foil sheet were placed with the fibrous side facing downward. The aluminium sheet was then peeled off and the prefabricated PDMS was placed on top of the fibrous membrane. The increased level of protein adsorption (eight times more than that on conventional TEPC) was observed in their model which has led to a lower LOD in immunoassays. This model can be useful for diagnostic studies based on antigen-antibody recognition.

Typical sealing methods can pose great challenges in terms of leakage of media due to weak sealing resulting from weak bonding between PDMS and fibres. Moreover, fibrous materials tend to be fragile and can crinkle under applied stated sealing methods. This hybrid technology requires more innovative sealing protocols for the optimization of bonding between PDMS and electrospun fibres.

A more innovative approach was used by Chen et al. which involved modularly integrating fibres in the microfluidic chip [223]. Fibres were electrospun on a polystyrene (PS) membrane which was then laser cut into rectangular inserts of similar dimensions to already made slots within a 3D-printed fluidic device. Fibres were immobilized on the PS membrane due to the fused edges resulting from the laser cut. They were cultured with desired cells before placing into the two slots. The media flowed through the space between the two inserts serving as the microfluidic channel which can easily be customized for adjusting the shear stress. This technology can also be cost-effective and efficient since failed cultures can be discarded without wasting the whole setup.

## 9 Concluding remarks and future outlook

Extensive research has been conducted to develop more biomimetic cell culture systems by recapitulating the physiological conditions. This includes developing multi-cell culture platforms, replacing cell lines with primary cells, improving the scaffold properties for a more physiologically relevant substrate, and introducing dynamic cell culture conditions and mechanical stimuli to the cells. OOCs technology, a new class of micro physiological *in vitro* models of human organs, can combine cells, chemical and physical environment and the microenvironment for developing the most physiologically relevant platform for research. Barrier tissues can be recapitulated by building this physiological biomimetic system on a microfluidic chip. The microenvironment of the organ in terms of tissue interfaces and mechanical stimulation can be recapitulated with this system. Although OOC technology has seen tremendous progress there are still numerous limitations which should be addressed in future OOC designs. They include preservation of the differentiation state of the cells over physiologically relevant culture durations, mimicking the composition of the cellular subsets in a dynamic manner, overcoming lack of connections with the other systems of the body (in particular vascular, nervous and immune systems), a general lack of representative microbiota, the issue of cell sourcing and the need for more physiologically relevant ECM structures.

There is an immediate need for more physiologically relevant membranes in OOCs. Porous flat membranes poorly represent the core structure of the barrier tissues *in vivo*. The integration of more biomimetic membranes in microfluidic devices is an emerging research area, with hydrogels and electrospun fibres being the most studied scaffolding materials due to their similarity to BMs.

Electrospinning holds great promise in the fabrication of micro/nanofibrous materials with sufficient porosity and a high surface area to volume ratio. However, their modulus is usually in the order of hundreds of MPa compared to only a few MPa for the stiffest BMs. Another challenge is the thickness of the membranes. The native BMs are ultrathin structures, the thickness of which is less than 1 μm. Handling thin electrospun membranes (less than 10 μm) is problematic. Electrospinning the membrane directly into a microfluidic channel can be a solution. Nevertheless, modular integration of electrospun fibres in the chip is preferred since the examination of the cultured cells is possible before the chip assembly which allows for the replacement of the scaffold in case of culture issues without discarding the whole set-up.

The integration of hydrogel materials in microfluidic chips is performed by injecting the hydrogel materials into the hollow microchannels followed by polymerization. However, photocuring can be a challenge. Alternatively, fabricating the channels from hydrogel bulk material can be used to introduce hydrogels to microfluidic chips. Although addressing the photocuring issue, the structures suffer from low structural integrity. The aforementioned techniques provide useful features correlated to the BM. Nevertheless, at this point, no scaffolding material can meet all the physiologic requirements of the naturally occurring BMs and this requires further research in this area.

One of the key advantages of microfluidic platforms is the ability to simulate blood flow and create an interface with vessel-like structures. In addition to allowing more efficient distribution of nutrients and removal of waste, this also provides an opportunity to simulate the migration of circulatory immune cells into tissue models and maintain a pool of tissue-resident immune cells such as monocyte-derived macrophages. While the inclusion of immune cells has been attempted in some OOCs, lack of immunocompetency remains one of the key limitations of many existing tissue models. As the field moves towards addressing this limitation, considering the physiological relevance of BM and ECM composition will be even more critical since both are known to have a significant impact on the functional properties of immune cells including migration, proliferation and differentiation. This should be another consideration in the future design and fabrication of physiologically relevant BM substitutes in OOCs.

## Declaration of competing interest

The authors declare that they have no known competing financial interests or personal relationships that could have appeared to influence the work reported in this paper.
